# TFAP2 paralogs regulate melanocyte differentiation in parallel with MITF

**DOI:** 10.1371/journal.pgen.1006636

**Published:** 2017-03-01

**Authors:** Hannah E. Seberg, Eric Van Otterloo, Stacie K. Loftus, Huan Liu, Greg Bonde, Ramakrishna Sompallae, Derek E. Gildea, Juan F. Santana, J. Robert Manak, William J. Pavan, Trevor Williams, Robert A. Cornell

**Affiliations:** 1 Interdisciplinary Graduate Program in Genetics, University of Iowa, Iowa City, Iowa, United States of America; 2 SDM-Craniofacial Biology, University of Colorado – Anschutz Medical Campus, Aurora, Colorado, United States of America; 3 Genetic Disease Research Branch, National Human Genome Research Institute, NIH, Bethesda, Maryland, United States of America; 4 Department of Anatomy and Cell Biology, University of Iowa, Iowa City, Iowa, United States of America; 5 Bioinformatics Division, Iowa Institute of Human Genetics, University of Iowa, Iowa City, Iowa, United States of America; 6 Bioinformatics and Scientific Programming Core, Computational and Statistical Genomics Branch, National Human Genome Research Institute, NIH, Bethesda, Maryland, United States of America; 7 Department of Biology, University of Iowa, Iowa City, Iowa, United States of America; Stanford University School of Medicine, UNITED STATES

## Abstract

Mutations in the gene encoding transcription factor TFAP2A result in pigmentation anomalies in model organisms and premature hair graying in humans. However, the pleiotropic functions of TFAP2A and its redundantly-acting paralogs have made the precise contribution of TFAP2-type activity to melanocyte differentiation unclear. Defining this contribution may help to explain why *TFAP2A* expression is reduced in advanced-stage melanoma compared to benign nevi. To identify genes with TFAP2A-dependent expression in melanocytes, we profile zebrafish tissue and mouse melanocytes deficient in *Tfap2a*, and find that expression of a small subset of genes underlying pigmentation phenotypes is TFAP2A-dependent, including *Dct*, *Mc1r*, *Mlph*, and *Pmel*. We then conduct TFAP2A ChIP-seq in mouse and human melanocytes and find that a much larger subset of pigmentation genes is associated with active regulatory elements bound by TFAP2A. These elements are also frequently bound by MITF, which is considered the “master regulator” of melanocyte development. For example, the promoter of *TRPM1* is bound by both TFAP2A and MITF, and we show that the activity of a minimal *TRPM1* promoter is lost upon deletion of the TFAP2A binding sites. However, the expression of *Trpm1* is not TFAP2A-dependent, implying that additional TFAP2 paralogs function redundantly to drive melanocyte differentiation, which is consistent with previous results from zebrafish. Paralogs *Tfap2a* and *Tfap2b* are both expressed in mouse melanocytes, and we show that mouse embryos with *Wnt1-Cre*-mediated deletion of *Tfap2a* and *Tfap2b* in the neural crest almost completely lack melanocytes but retain neural crest-derived sensory ganglia. These results suggest that TFAP2 paralogs, like MITF, are also necessary for induction of the melanocyte lineage. Finally, we observe a genetic interaction between *tfap2a* and *mitfa* in zebrafish, but find that artificially elevating expression of *tfap2a* does not increase levels of melanin in *mitfa* hypomorphic or loss-of-function mutants. Collectively, these results show that TFAP2 paralogs, operating alongside lineage-specific transcription factors such as MITF, directly regulate effectors of terminal differentiation in melanocytes. In addition, they suggest that TFAP2A activity, like MITF activity, has the potential to modulate the phenotype of melanoma cells.

## Author summary

Identifying the elements and structure of the gene regulatory network governing melanocyte differentiation may yield insight into the mechanisms of pigmentation diseases and melanoma progression. Pigmentation is abnormal in *Tfap2a* mutants, but deciphering the exact role of TFAP2A in the network has been complicated by pleiotropic requirements for TFAP2A during development and the redundant function of TFAP2 paralogs in melanocytes. In this study, we find that TFAP2A directly regulates genes involved in melanocyte differentiation and melanin synthesis by binding at both promoters and enhancers associated with these genes. Furthermore, we report evidence that TFAP2A shares many targets with the melanocyte “master regulator” MITF. These findings indicate that TFAP2A drives melanocyte differentiation in parallel with MITF and affects the net pro-differentiation activity that is lost in melanoma.

## Introduction

Melanocytes are responsible for pigment deposition in skin and hair follicles, and the dysregulation of melanocyte differentiation underlies both pigmentation disorders and melanoma. Because melanocytes are dispensable for life, the melanocyte lineage can also serve as a model for investigation of developmental processes important in all cell types. Many transcription factors and other regulatory molecules that drive melanocyte development have been identified through genetic analyses of patients with congenital pigmentation disorders, including piebaldism (*SNAI2*) [1], Waardenburg syndrome types I and III (*PAX3*) [2], Waardenburg syndrome type II (*SNAI2*, *MITF*, *SOX10*) [3–5], and Waardenburg-Shah syndrome (*EDN3*/*EDNRB*, *SOX10*) [6,7]. Epistasis experiments in model organisms have begun to assemble these genes into functional hierarchies, also called gene regulatory networks (GRNs), that govern specific processes in the development of melanocytes from the neural crest. For example, work in mouse and chick indicates that during melanocyte lineage specification, PAX3 and SOX10 activate expression of *MITF* [8] and FOXD3 represses it [9–11], while SOX2 and MITF appear to cross-regulate expression of each other [12,13]. A recent integrated analysis of ChIP-seq and expression profile data in mouse found that SOX10 directly activates expression of many genes implicated in melanocyte differentiation, and suppresses those that promote pluripotency [14]. Similarly, MITF ChIP-seq and enhancer deletion studies in human cell lines have shown that in addition to its role in melanocyte fate specification, MITF directly stimulates the expression of many genes encoding effectors of melanin synthesis, including *Dopachrome Tautomerase* (*DCT*) [15, reviewed in 16,17,18]. Because mice with loss-of-function mutations in *Mitf* lack melanocytes, and ectopic expression of MITF activates expression of melanin synthesis genes in heterologous cell types, MITF is considered a “master regulator” of melanocyte development [19–21]. MITF activity has also been described as a rheostat that regulates melanoma phenotype by driving senescence at low levels, an invasive phenotype at mid-levels, and melanocyte proliferation and differentiation at higher levels [22]. Continued exploration of the GRNs controlling melanocyte differentiation will add to the value of the melanocyte as a model cell type, and may also guide the design of differentiation-promoting therapies in melanoma.

Mutations in *Transcription Factor Activating Enhancer-Binding Protein 2 Alpha* (*TFAP2A*) result in pigmentation phenotypes similar to those caused by mutations in established members of the melanocyte differentiation GRN. In humans, a variety of missense mutations in *TFAP2A* cause branchio-oculo-facial syndrome, which frequently includes premature hair graying due to dysregulation of melanocyte stem cells [23]. Mice with *Wnt1-Cre*-mediated deletion of *Tfap2a* in neural crest usually die from exencephaly, but rare surviving animals exhibit a white belly spot analogous to the phenotype of mutants heterozygous for a null allele of *Kit* [24], which is thought to be a direct target of TFAP2A [25,26]. We also observe a greater-than-additive belly-spot phenotype in *Tfap2a* and *Kit* double heterozygous mice, signifying a genetic interaction between these genes (TW, unpublished observations). In zebrafish embryos homozygous for strong loss-of-function alleles of *tfap2a*, melanocytes are fewer in number and exhibit reduced migration relative to melanocytes in wildtype embryos [27–29]. This phenotype resembles zebrafish *kita* mutants, and there is also evidence of genetic interaction between *tfap2a* and *kita* in zebrafish [29]. However, zebrafish *tfap2a* mutants also have a phenotype of delayed melanization that is not present in zebrafish *kita* mutants [27–29], and we previously showed that *tfap2a* and its paralog *tfap2e* are cell-autonomously required for melanocyte differentiation in zebrafish [30]. These phenotypes imply that TFAP2A contributes to the GRN governing melanocyte migration, possibly upstream of *KIT*, as well as to a GRN governing melanocyte differentiation by mediating expression of unknown targets.

The precise contribution of TFAP2A to the melanocyte differentiation GRN has been obscured by pleiotropic functions of TFAP2A and its redundantly-acting paralogs during earlier steps in neural crest development. TFAP2A belongs to a family of five paralogs, TFAP2A-E, of which all but TFAP2D have an identical sequence binding preference [reviewed in 31]. In all species thus far analyzed, TFAP2A and one or more additional TFAP2 paralogs with potential for redundant activity are expressed in the neural plate border, premigratory neural crest, and melanocytes, but the identity of the additional paralogs varies among species. Zebrafish melanocytes express *tfap2a*, *tfap2c*, and *tfap2e* [32], and embryos depleted of both *tfap2a* and *tfap2e* display a greater-than-additive reduction in both melanocyte number and pigmentation compared to embryos depleted of either gene alone [30]. However, it has not yet been possible to examine the consequence of removing all three Tfap2 paralogs in melanocytes due to another example of redundancy, the lack of neural crest in zebrafish depleted of both Tfap2a and Tfap2c [33,34]. This is also true in mouse, where *Tfap2a* and *Tfap2b* are expressed in early neural crest [35] as well as the melanocyte lineage, resulting in almost complete loss of migrating trunk neural crest prior to specification of the melanocyte lineage in *Tfap2a/Tfap2b* double mutants [36]. Thus, the specific contributions of these factors to the GRN governing melanocyte differentiation have not been thoroughly evaluated.

In this study, we investigate the role of TFAP2A in melanocyte differentiation, utilizing the different advantages of zebrafish, mouse, and cell line models. While pigmentation is clearly reduced in zebrafish *tfap2a* mutants, *mitfa* expression levels appear to be normal in the remaining melanocytes [30]. Likewise, it was reported that in 501mel melanoma cells depleted of *TFAP2A*, the expression levels of *MITF* were unchanged compared to control cells, while expression of *TYR*, encoding the rate limiting enzyme of melanin synthesis, was decreased [37]. Pigmentation phenotypes in zebrafish *tfap2a* mutants are therefore unlikely to be an effect of altered Mitf expression levels. However, MITF activity is regulated by post-translational modifications [38–40], and the expression of enzymes mediating these modifications may depend on TFAP2A. Alternatively, TFAP2A and MITF could directly co-regulate expression of melanocyte differentiation effectors. In support of this model, there is evidence that both proteins regulate expression of *CDKN1A/p21* [41,42] and *IRF4* [37]. Furthermore, a recent integrative analysis of chromatin mark data in 111 cell types indicated that enhancers active in melanocytes are enriched in the TFAP2A binding site [43], although other, non-TFAP2 family transcription factors may bind similar sites. Co-occupancy of enhancers by MITF and TFAP2A was also reported in human melanoma cell lines [44]. However, melanomas often have reduced TFAP2A expression, accompanied by methylation of the *TFAP2A* promoter, and so are not ideal systems to study the role of TFAP2A in normal melanocyte development and function [26,45,46]. Here we examine the relationship between TFAP2A and MITF in the context of melanocytes using a combination of molecular, genetic, and bioinformatic analyses in human, mouse, and zebrafish systems. The results confirm that TFAP2A frequently co-occupies regulatory elements with MITF, identify genes underlying pigmentation phenotypes in model organisms and patients with *TFAP2A* mutations, and reveal TFAP2A as a candidate locus to modify diseases associated with MITF, including melanoma.

## Results

### TFAP2A is required for normal expression of melanocyte differentiation genes

**Fig 1:**
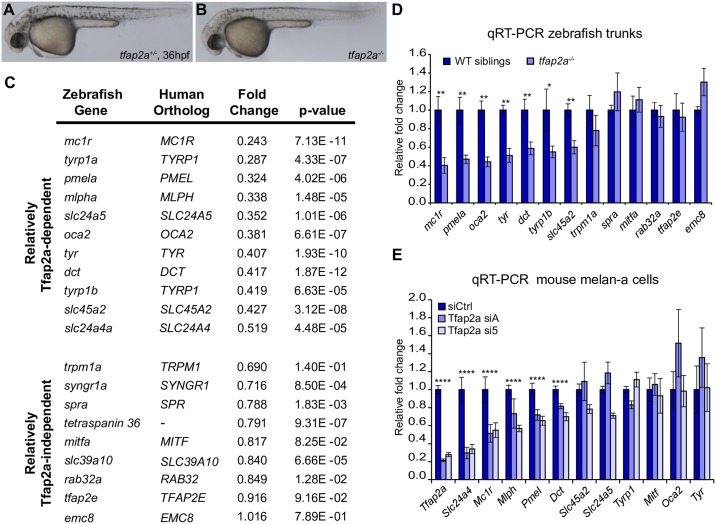
Melanocyte differentiation genes are sensitive to the loss of TFAP2A. (A, B) Lateral images of *tfap2a*^*+/-*^ and *tfap2a*^*-/-*^ mutant zebrafish at 36 hpf. *tfap2a*^*-/-*^ mutants have fewer melanocytes and delayed pigmentation relative to their wildtype or *tfap2a*^*+/-*^ siblings. (C) Fold changes calculated from microarray expression levels for 20 zebrafish melanocyte genes. Top: 11 genes with expression levels between 0.2–0.55-fold in *tfap2a*^*-/-*^ mutants compared to WT siblings. Bottom: 9 genes with expression levels reduced no further than the number of melanocytes in *tfap2a*^*-/-*^ mutants. (D) qRT-PCR validation of zebrafish microarray results for 13 melanocyte genes (Mann-Whitney U, *p<0.05, **p<0.01). (E) qRT-PCR validation of microarray results for 12 melanocyte genes in mouse melan-a cells transfected with control siRNA or each of two siRNAs targeting *Tfap2a* (siA, si5). Significance evaluated only when both siA and si5 altered gene expression in the same direction relative to siCtrl (one-way ANOVA with Bonferroni correction, ****p<0.0001).

Zebrafish homozygous for a strong loss-of-function mutation in *tfap2a* (i.e., *lockjaw* allele, hereafter *tfap2a*^*-/-*^ mutants) lack detectable anti-TFAP2A immunoreactivity [28,47] and exhibit approximately one-third reduction of embryonic melanocytes, impaired melanocyte migration, and delayed melanization relative to wildtype and *tfap2a*^*+/-*^ siblings (Fig 1A and 1B) [27,30,48]. In order to better characterize the melanization phenotype, we compared *tfap2a*^*-/-*^ mutants to *tfap2a*^*+/-*^ siblings over a ten-hour period, starting with the first emergence of melanocytes around 28 hours post fertilization (hpf) (S1 Fig). While melanocytes are initially pale in both genotypes, individual melanocytes in the *tfap2a*^*+/-*^ siblings pigmented more quickly than individual melanocytes in *tfap2a*^*-/-*^ mutants. This supports earlier evidence that, in addition to reduced numbers and migration, melanocytes in *tfap2a*^*-/-*^ mutants have defects in differentiation [30].

To extend previous analyses of gene expression in the melanocytes of *tfap2a*^*-/-*^ mutants [30,34,48,49], we generated expression profiles of *tfap2a*^*-/-*^ mutant zebrafish and their wildtype siblings at 36 hpf. Prior to harvesting RNA, we decapitated animals to eliminate the retinal pigmented epithelium, which appears to be normally pigmented in *tfap2a*^*-/-*^ mutants (Fig 1A and 1B). Generating cDNA and probing microarrays revealed that 2,337 unique Ensembl transcripts (corresponding to 2,324 genes) are differentially expressed in the trunks of *tfap2a*^*-/-*^ mutants versus siblings (FDR p<0.05). Of these, the expression levels of 124 transcripts in *tfap2a*^*-/-*^ mutants are decreased to ≤0.7-fold of wildtype levels and 358 transcripts are increased to ≥1.25-fold (expression profile in S1 Table). We referred to zebrafish gene expression patterns at an online database (ZFIN) to identify 19 genes annotated as “melanoblast,” “melanocyte,” or “pigment cell” [50]. An additional gene, *slc24a4a*, is annotated as “neural crest,” but is expressed in a pattern resembling that of *dct* [51]. Most of these 20 genes encode proteins that have known roles in melanocyte differentiation (Fig 1C, S2 Table). In *tfap2a*^*-/-*^ mutants, 11 of these genes were expressed between 0.2- to 0.55-fold of wildtype levels, a much greater fraction of melanocyte genes than expected by chance (hypergeometric test, p<0.0001), and were therefore considered Tfap2a-dependent (Fig 1C). The levels of several others, including *trpm1a*, were not significantly changed or were reduced by no more than expected from the one-third decrease in melanocyte cell number, and we considered these to be Tfap2a-independent. qRT-PCR validation of microarray results confirmed that expression of multiple pigmentation genes depends on Tfap2a in zebrafish (Fig 1D).

Because TFAP2A is expressed in multiple tissues, including the skin where melanocytes reside, the Tfap2a-dependent gene expression in zebrafish melanocytes could reflect an indirect requirement for Tfap2a rather than a cell-autonomous one. To clarify this issue, we conducted a microarray expression profile of immortalized mouse melanocytes (melan-a cells) [52] depleted of *Tfap2a*. We identified two independent siRNAs that reduced expression of *Tfap2a* to below 0.25-fold of the level in cells transfected with control siRNAs. Comparison of microarray profiles showed that the expression levels of 30 genes were consistently decreased (≤0.7-fold) and the expression levels of 38 genes were consistently increased (≥1.4-fold) in cells transfected with either *Tfap2a*-targeted siRNA relative to control siRNAs (S3 Table). Among the 30 genes with decreased expression, *Mc1r* and *Slc24a4* are implicated in pigmentation. qRT-PCR verified significant reduction of these two genes, as well as *Dct*, *Mlph*, and *Pmel*, which narrowly missed the threshold cut-off in the array analysis (Fig 1E). Of note, several of the other genes with decreased expression upon knockdown of *Tfap2a* have been reported in the literature as activated (*Aldh1a3*, *Igf2bp1*, *Ephb2*, *Pbk*) or downregulated (*Qpct*, *Wfdc1*) in melanoma [53–59]. In summary, expression of a subset of melanocyte differentiation genes, including *Slc24a4*, *Mc1r*, *Mlph*, *Pmel*, and *Dct*, was TFAP2A-dependent in both mouse melanocytes and zebrafish trunks, while expression of *Mitf* orthologs was TFAP2A-independent. Certain pigmentation genes, including *oca2* and *slc45a2*, appeared Tfap2a-dependent in zebrafish but not in mouse melanocytes, perhaps reflecting a more thorough depletion of Tfap2a in the former, or potentially distinct homeostatic mechanisms induced by siRNA versus gene mutation. Nonetheless, these results indicate that TFAP2A, whether directly or indirectly, regulates the expression of genes involved in melanocyte differentiation.

### TFAP2A occupancy in mouse and human melanocytes

To determine the direct transcriptional targets of TFAP2A in melanocytes, we conducted chromatin immunoprecipitation followed by high-throughput sequencing (ChIP-seq) in mouse melan-a cells (two replicates) and in human primary melanocytes (one replicate). Anti-TFAP2A immunoreactivity is strong and concentrated in the nucleus of human primary melanocytes (S2A Fig) but appears weaker and more diffuse in several melanoma cell lines (S2B–S2E Fig), consistent with a reduction of *TFAP2A* RNA levels in melanoma [45]. ChIP-seq detected 16,305 TFAP2A-bound loci in mouse melanocytes, and 13,690 TFAP2A-bound loci in human melanocytes (hereafter, TFAP2A peaks). *De nov*o motif analysis [60] of sequences precipitated by the anti-TFAP2A antibody from mouse or human melanocytes revealed that a known TFAP2A binding site (MA0003.2, JASPAR) is strongly enriched and tends to be centrally located within peaks (S3A and S3C Fig). Anti-TFAP2A ChIP followed by quantitative PCR (ChIP-qPCR) confirmed TFAP2A binding enrichment for selected peaks at genes of interest in mouse or human melanocytes, as well as in the M21 melanoma cell line, which has detectable TFAP2A expression (S3B, S3D and S3E Fig).

Published comparisons of ChIP-seq results for a given transcription factor in mouse and human melanocytes have suggested rapid divergence of binding events during evolution [61–63]. Consistent with these studies, we found that only about 11% of TFAP2A peaks in human primary melanocytes coincide with the orthologs of TFAP2A peaks lifted over from mouse (S4 Table), and conversely, about 9% of the TFAP2A peaks identified in mouse melanocytes coincide with peaks lifted over from human (S5 Table). TFAP2A peaks shared between species are enriched near promoters (S6 Table). In contrast to the modest concordance of peaks and orthologous sequences, the concordance of genes associated with TFAP2A peaks in the two species is very high, as shown below.

### Relationship between TFAP2A occupancy and defined regulatory elements

**Fig 2:**
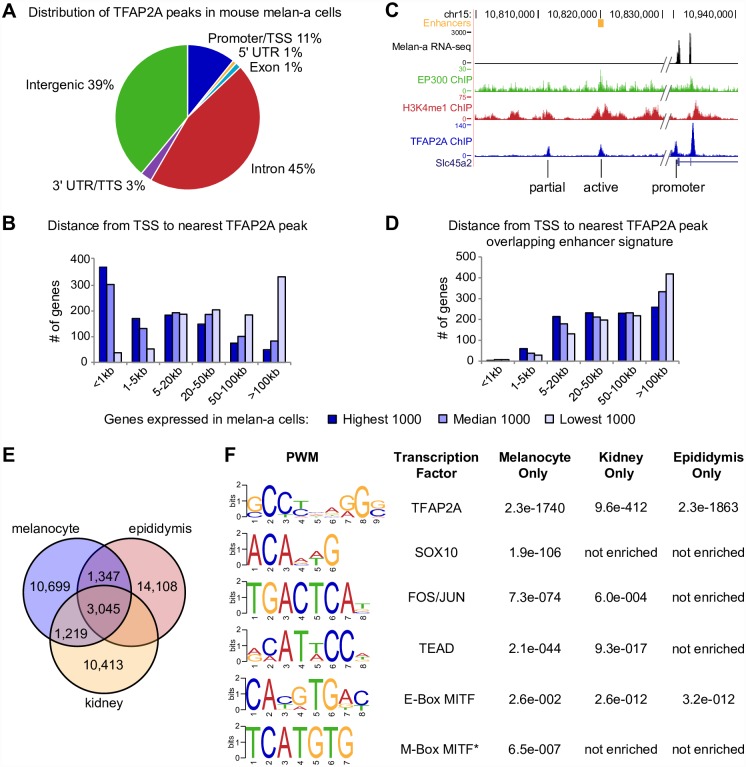
TFAP2A binds active enhancers and promoters in mouse melanocytes. (A) Pie chart showing distribution of mouse TFAP2A peaks with respect to genomic features. TSS, transcription start site; TTS, transcription termination site. (B) Distance from TSS to the nearest TFAP2A peak for genes in three expression categories: highest 1000, median 1000, or lowest 1000. Promoter-proximal TFAP2A peaks are enriched at highly expressed genes (RNA-seq on mouse melan-a cells at GSE87051). (C) Examples of active enhancer signatures defined by H3K4me1 peaks flanking a p300 peak [65], and partial enhancer signatures, overlapping TFAP2A peaks upstream of the melanocyte differentiation gene *Slc45a2*. (D) Distance from TSS to the nearest TFAP2A peaks that overlap the active enhancer signature for genes in three expression categories: highest 1000, median 1000, or lowest 1000. (E) Overlap of TFAP2A ChIP-seq peaks in mouse melanocytes with published TFAP2A ChIP-seq peaks in mouse kidney and epididymis cells [68]. 34% of melanocyte peaks were shared with one or both of the other cell types. (F) MEME-ChIP analysis of unique peaks from each cell type. Melanocyte-unique peaks are significantly enriched for SOX10 and M-Box MITF binding motifs, while kidney-unique and epididymis-unique peaks are not. *All motifs shown are a result of *de novo* MEME-ChIP enrichment analysis except the M-Box, which we specifically searched using the Analysis of Motif Enrichment (AME) tool [109].

In both mouse and human melanocytes, TFAP2A peaks are more likely to be found within genes, including introns, than in intergenic regions (Fig 2A, S4A Fig). Overall, genes with higher expression in melanocytes are enriched for promoter-proximal peaks of TFAP2A (mouse GSE87051, human GSM958174 [64]) (Fig 2B, S4B Fig). To assess patterns of TFAP2A binding at enhancers, we compared the TFAP2A ChIP-seq data from mouse melan-a cells to a published profile of candidate enhancers also in these cells, which were defined by H3K4me1 peaks flanking a p300 peak [65]. Remarkably, 70% (1,752 of 2,489) of enhancer elements marked in this way overlap with a TFAP2A peak (hypergeometric test, p<0.0001). Conversely, about 10% of TFAP2A peaks are fully marked as enhancers, while another 35% are partially marked (i.e., either overlapping p300 or flanked by at least one H3K4me1 peak). Fig 2C illustrates this pattern upstream of the melanocyte differentiation gene *Slc45a2*, as well as a TFAP2A peak at the promoter (additional examples in S5A–S5F Fig). In agreement with estimates that the median distance between promoters and cis-acting enhancers is 15kb, genes with high expression are enriched for a TFAP2A peak overlapping the active enhancer signature at a distance of 5–50kb from the TSS (Fig 2D) [66]. However, the promoter-proximal TFAP2A peaks are just as likely to possess a TFAP2A binding site as the promoter-distal peaks (about 50% in both cases). This is in contrast to observations from mouse chondrocytes, where promoter-distal SOX9 peaks are more likely to contain a SOX9 binding site than promoter-proximal ones [67]. Altogether, the binding profile of TFAP2A indicates that it acts at both enhancers and promoters of melanocyte genes.

### TFAP2A-bound chromatin elements are enriched for distinct binding motifs in different cell types

Because TFAP2A is widely expressed, the extent to which TFAP2A peaks would be melanocyte-specific was unclear. We found that about 15% of TFAP2A peaks in mouse melanocytes overlap those reported in either primary mouse kidney or primary mouse epididymis cells (but not both), and 19% are shared by all three cell types [68] (Fig 2E). MEME-ChIP analysis [69] showed that melanocyte-unique TFAP2A peaks are enriched for the binding motifs of transcription factors active in melanocytes, including SOX10, FOS/JUN, TEAD, and the M-box binding site for MITF [70] (Fig 2F). These transcription factors are also highly enriched in candidate melanocyte enhancers [65]. Kidney-unique and epididymis-unique peaks showed less significant or no enrichment for these binding motifs, although the E-box, recognized by MITF and many other bHLHZip transcription factors, was enriched in all three cell types. The enrichment of binding sites for melanocyte transcription factors like SOX10 and MITF in melanocyte-unique peaks suggests that TFAP2A binds cell-type specific loci in addition to generic ones; binding to any particular locus is presumably a function of cofactor availability and chromatin accessibility [reviewed in 71].

### Many TFAP2A-dependent melanocyte genes are direct targets of TFAP2A

**Fig 3:**
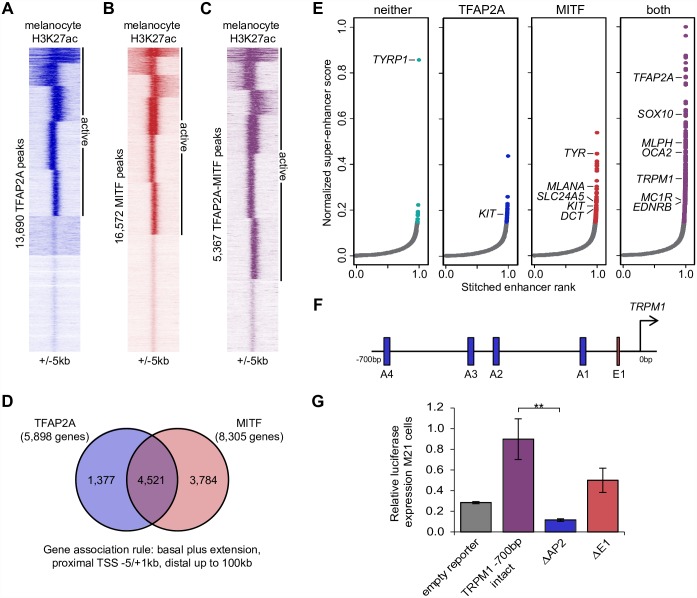
TFAP2A peaks are associated with genes involved in pigmentation. (A-C) Density-based clustering of H3K27ac signal at (A) TFAP2A peaks, (B) MITF peaks, and (C) TFAP2A peaks that overlap MITF peaks in human melanocytes (H3K27ac data from GSM1127072 [64]), MITF peaks from [18]). (D) Overlap between genes associated with active TFAP2A peaks and genes associated with active MITF peaks in human melanocytes. (E) Typical enhancers (gray) and super-enhancers (colored) in human melanocytes that overlap neither TFAP2A nor MITF peaks, TFAP2A peaks only, MITF peaks only, or both TFAP2A and MITF peaks. Labels identify melanocyte genes of interest. (F) Diagram of the *TRPM1* -700 bp promoter element depicting the positions of four TFAP2A binding sites (A1–A4) and the previously reported E-box 1 MITF binding site (E1). (G) Luciferase assays in M21 melanoma cells. Deletion of all four TFAP2A binding sites (ΔAP2) significantly reduced reporter activity compared to the intact *TRPM1* -700bp element (Student’s t-test, **p = 0.01).

We used Genomic Regions Enrichment of Annotations Tool (GREAT) [72] to identify genes associated with TFAP2A peaks in mouse and human melanocytes, with an assignment rule of basal promoter plus 100kb distal, and found that these genes are enriched for ontology terms relevant to melanocyte differentiation, including “pigmentation”, “melanosome”, and “melanoma” (S6A and S6B Fig). However, TFAP2A can both activate and repress gene expression [73]. To find genes likely to be activated by TFAP2A, we integrated our TFAP2A ChIP-seq with H3K27ac ChIP-seq data from human melanocytes (GSM1127072 [64]) and mouse melanocytes [14], as H3K27ac marks active regulatory elements [reviewed in 74,75]. We found that 55% of TFAP2A peaks in human melanocytes and 58% of TFAP2A peaks in mouse melanocytes either overlap H3K27ac peaks or are flanked by H3K27ac peaks, and we refer to them hereafter as active TFAP2A peaks (human Fig 3A, mouse S6C Fig, S7 Table). Analysis with GREAT revealed that genes associated with active TFAP2A peaks include 15 of the 30 genes with significantly decreased expression in *Tfap2a*-depleted mouse melanocytes, among them *Mc1r* and *Slc24a4* (S8 Table). Similarly, orthologs of eight out of ten Tfap2a-dependent melanocyte genes in zebrafish *tfap2a*^*-/-*^ mutants (excepting *slc24a5* and *tyr*) are associated with active TFAP2A peaks in mouse or human melanocytes. These genes are candidates to be direct transcriptional targets of TFAP2A, suggesting that the phenotype of delayed melanization in zebrafish *tfap2a*^*-/-*^ mutants can be explained in part by a direct effect on certain melanocyte differentiation effector genes (e.g., *dct*, *mlpha*, *mc1r*, and *pmela*), and indirect regulation of others (e.g., *slc24a5* and *tyr*).

### TFAP2A and MITF co-occupy regulatory elements associated with pigmentation genes

The presence of TFAP2A peaks at a majority of active melanocyte enhancers, as well as the enrichment of the MITF binding motif in TFAP2A peaks, implies that TFAP2A binds many of the same regulatory elements as MITF in melanocytes. To evaluate overlap between TFAP2A and MITF across the genome, we compared our human TFAP2A ChIP-seq results to a set of 16,572 MITF ChIP-seq peaks also from human primary melanocytes [18]. 5,367 (39%) of TFAP2A peaks are shared with MITF, overlapping by at least one base pair. Integrating MITF and H3K27ac ChIP-seq data yielded 61% of MITF peaks (Fig 3B) and 76% of TFAP2A/MITF shared peaks (Fig 3C) that overlap, or lie between, H3K27ac peaks and are thus considered to be active. Using GREAT, we found that about 77% of the genes associated with active TFAP2A peaks are also associated with active MITF peaks, a highly significant overlap (hypergeometric test, p<0.0001) (Fig 3D). Furthermore, 79% of these genes are associated with active TFAP2A/MITF shared peaks, suggesting that TFAP2A and MITF are co-bound at many, but not all, shared targets. GO term analysis [76,77] revealed that the subset of genes associated with both TFAP2A and MITF peaks are enriched for the terms “melanosome” and “pigment granule” (p = 9.08E-07), as well as “DNA repair” (p = 5.98E-08), “mitotic cell cycle process” (p = 5.59E-09), “regulation of cell proliferation” (p = 2.40E-03), and “regulation of cell differentiation” (p = 2.60E-03) (all p-values Bonferroni corrected, S9 Table). This supports a regulatory role for TFAP2A not only in differentiation, but across other categories of genes proposed to be regulated by MITF in melanocytes and melanoma, as with the MITF rheostat [22].

To assess the overlap between targets of TFAP2A and MITF with respect to pigmentation, we focused on a list of 170 genes that cause coat color phenotypes in mice [78], adding *TRPM1* based on its role in the coat color phenotype of appaloosa horses [79–82]. Orthologs of 97 genes on this list are associated with active TFAP2A peaks in human melanocytes and/or active TFAP2A peaks in mouse melanocytes (Table 1, asterisks). Of these, 72 genes are also associated with active MITF peaks (Table 1), 46 being active shared TFAP2A/MITF peaks (Table 1, bold). We then examined overlap of MITF and TFAP2A binding at clusters of closely spaced enhancers, sometimes called stretch or super-enhancers (SEs) [83], that are linked to cell type-specific gene expression [84,85]. Following published methods, we used H3K27ac data to identify 652 SEs in human primary melanocytes [85] (S7 Fig). Of these, 530 (81%) are bound by both MITF and TFAP2A (Fig 3E, S10 Table). Interestingly, genes involved in melanocyte differentiation, including those encoding proteins expressed in the melanosome, are associated with SEs bound by MITF only (e.g. *TYR*, *MLANA*, *SLC24A5*, *DCT*) and SEs bound by both MITF and TFAP2A (*MLPH*, *OCA2*, *TRPM1*, *MC1R)*. Exceptions to this pattern include *KIT*, which is associated with one SE bound solely by TFAP2A and one SE bound solely by MITF, and *TYRP1*, which is associated with an SE bound by neither (Fig 3E). Taken together, these results show that TFAP2A and MITF bind regulatory elements associated with melanocyte differentiation effectors. Notably, 409 (63%) of all SEs are bound by TFAP2A peaks that overlap with MITF peaks. It remains to be determined whether TFAP2A and MITF exhibit cooperative binding at these loci.

**Table 1:** Mouse coat color genes associated with peaks of TFAP2A and MITF that overlap active enhancer marks (H3K27ac).

	TFAP2A		Both			MITF
**Melanosome**	MYO7A*	SHROOM2*	***AP3B1***	***HPS6***	***PMEL***	*AP3D1*
			DCT*	***LYST***	***RAB38***	*BLOC1S3*
			DTNBP1*	***MLPH***	RABGGTA*	*HPS1*
			*GPNMB*	***MREG***	SLC45A2*	*RAB27A*
			HPS3*	MYO5A*	TYRP1*	*SLC24A5*
			***HPS4***	***OCA2***	***VPS33A***	*TRAPPC6A*
			HPS5*			*TYR*
**Eu-/pheo- melanin**	*EDARADD*	POMC*	***ATRN***	***MGRN1***	***SMARCA5***	*GGT1*
			***MC1R***	***OSTM1***	***SMCHD1***	
			***MCHR1***			
**Development**	ADAMTS20*	KITLG*	*ADAM17*	*GPR161*	*PHACTR4*	*EGFR*
	*ARCN1*	MITF*	*APC*	***ITGB1***	PYGO1*	*FOXD3*
	*DOCK7*	*NF1*	***BMPR1A***	***JMJD6***	RB1*	*FZD4*
	*EED*	NOTCH2*	BRCA1*	*KIT*	***SEMA3C***	
	FOXN1*	*RECQL4*	*CITED1*	LEF1*	***SEMA4A***	
	*GNA11*	S1PR2*	***DPH1***	***MAP2K1***	***SNAI2***	
	GRLF1*	*SUFU*	***ECE1***	MCOLN3*	***SOX10***	
	HELLS*	UNC119*	***EDNRB***	***MYC***	***TFAP2A***	
			***GAS1***	*NOTCH1*	***TIMP3***	
			***GLI3***	*PAX3*	***TRAF6***	
			***GNAQ***	***PDGFB***	ZBTB17*	
			***GNPAT***			
**Systemic effects**	*ATOX1*	*FAS*	BCL2*	***PDPK1***	***RXRA***	*ATP7A*
	*ATP7B*	RBP1*	***CASP3***	***POLG***	***TRPM1***	*RPS20*
	DST*		***HS2ST1***	POLH*	***TRPM7***	*SLC31A1*
			***OAT***	***RPL24***	*VLDLR*	

* = associated with a TFAP2A peak in mouse but not human melanocytes

**bold** = TFAP2A and MITF peaks associated with these genes overlap

While several of the pigmentation genes associated with active TFAP2A and MITF peaks showed TFAP2A-dependent expression in both zebrafish and mouse, we also noted many apparently TFAP2A-independent genes on this list. One possible explanation is that the presence of a TFAP2A peak does not signify contribution of TFAP2A to the activity of a given regulatory element. Alternatively, activity of a redundantly-expressed TFAP2 paralog may compensate for the loss of TFAP2A. To rule out the first possibility, we focused on the gene *TRPM1*, which has a promoter-proximal TFAP2A peak in both mouse and human melanocytes but is expressed at high levels in melanocytes in a TFAP2A-independent manner. In addition, expression of *TRPM1* is a sensitive readout of MITF activity levels [79], and a minimal *TRPM1* promoter, which has an MITF peak in melanoma cells [17] and primary melanocytes [18], has activity in melanoma cells that is lost upon deletion of the MITF binding sites [86]. We engineered the *TRPM1* promoter into a vector suitable for quantitative luciferase reporter assays and created variants with mutations in either the E-Box MITF binding site shown previously to be most important for promoter activity (ΔE1) [86], or four predicted TFAP2 binding sites (ΔAP2) (Fig 3F). These constructs, together with a control vector, were transfected into M21 melanoma cells, which express TFAP2A (S2C Fig). The intact *TRPM1* promoter drove much higher luciferase expression than the empty reporter vector, while the ΔE1 promoter variant drove about 50% of intact, and the ΔAP2 variant drove about 10% of intact (p = 0.01) (Fig 3G). These results show that TFAP2A directly activates the *TRPM1* promoter, supporting the hypothesis that other TFAP2 paralogs are able to compensate for the absence of TFAP2A at certain melanocyte genes.

### Mouse *Tfap2a / Tfap2b* double conditional mutants are depleted of melanocytes

**Fig 4:**
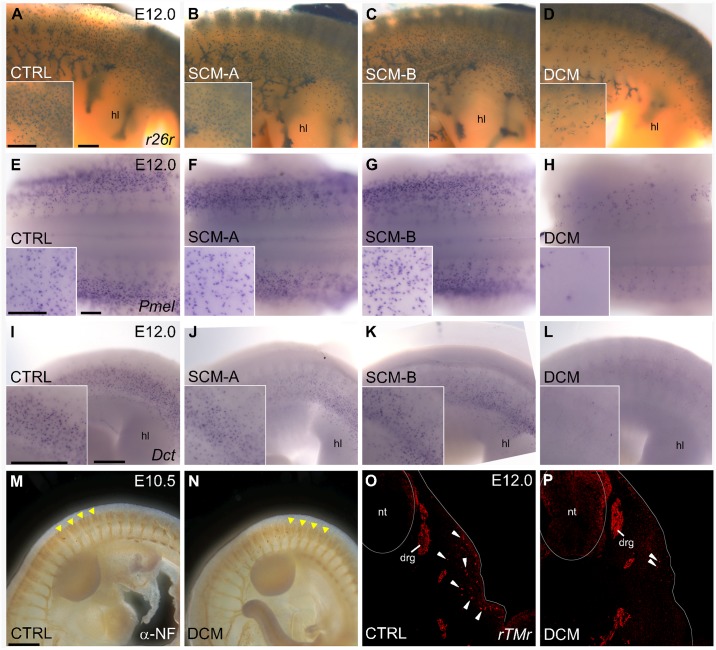
TFAP2A and TFAP2B redundantly regulate murine melanocyte development. (A-D) Lateral views of E12.0 control (A), *Tfap2a* SCM (B), *Tfap2b* SCM (C), or *Tfap2a/Tfap2b* DCM (D) mouse embryos processed for β-galactosidase (β-gal) staining. β-gal+ cells are a result of recombination of the *r26r*-allele by the *Wnt1-Cre* transgene, labeling neural crest cells and derivatives. (E-L) *In situ* hybridization expression patterns for *Pmel* (E-H, dorsal views) or *Dct* (I-L, lateral views) in control (E, I), *Tfap2a* SCM (F, J), *Tfap2b* SCM (G, K), or *Tfap2a/Tfap2b* DCM (H, L) E12.0 mouse embryos. Insets in (A-L) show higher magnification images just above the hindlimb in a similar viewing plane as the low magnification image. (M, N) Lateral views of the trunk processed for ɑ-neurofilament immunoreactivity in an E10.5 control (M) or *Tfap2a*/*Tfap2b* DCM (N), revealing developing dorsal root ganglia (a subset labeled with arrowheads). (O, P) Immunofluorescence of cryosections (cross-sectional plane at the level of the hindlimb) of an E12.0 control (O) or *Tfap2a*/*Tfap2b* DCM (P), containing a Tomato-reporter (*rTMr*) allele labeling neural crest cells and derivatives (arrowheads highlight a subset of melanocytes in the ventrolateral pathway). Abbreviations: DCM, double conditional mutant; drg, dorsal root ganglia; hl, hindlimb; nt, neural tube; SCM, single conditional mutant (A = *Tfap2a* or B = *Tfap2b*). Scale bars = 250μM, except M, N = 500μM.

Testing whether TFAP2 paralogs function redundantly in melanocyte development requires simultaneous depletion of all such paralogs expressed in melanocytes. In mouse melanocytes, *Tfap2a* and *Tfap2b* have the highest and second highest expression, respectively, while *Tfap2c* and *Tfap2e* are undetectable [87,88]. To determine whether *Tfap2* paralogs function redundantly in murine melanocyte development, we generated double conditional mutants (DCM) using a previously published *Wnt1-Cre* transgenic line [89] and conditional alleles of *Tfap2a* [24] and *Tfap2b* (EVO and TW, in preparation). We then utilized two approaches to assess melanocyte development in DCM embryos, corresponding single conditional mutant (SCM) embryos, and control embryos. First, embryos in which the Rosa26-reporter (*r26r*)-allele [90] was also incorporated were dissected at embryonic day 12.0 (E12.0) and subsequently stained for β-galactosidase (β-gal) activity. The *r26r-*allele used in combination with the *Wnt1-Cre* transgene results in β-gal positive staining of premigratory neural crest cells and subsequent derivatives (Fig 4A–4D, S8A–S8D Fig). β-gal-positive melanoblasts and corresponding melanocytes migrate ventrolaterally from the dorsal neural tube and can be identified by their position just below the developing surface ectoderm, most easily observed dorsal to the hindlimb. Examination of control (Fig 4A), *Tfap2a* SCM (Fig 4B), and *Tfap2b* SCM (Fig 4C) embryos revealed roughly equivalent numbers of stained cells with a similar distribution. In contrast, *Tfap2a/Tfap2b* DCM embryos have many fewer β-gal-positive cells in this location (Fig 4D). Second, embryos were processed for *in situ* hybridization with *Pmel* [91] and *Dct* [92,93] riboprobes, detecting melanoblasts and differentiated melanocytes (Fig 4E–4L). As with the *r26r* experiments, this staining labeled similar numbers of *Pmel*-positive and *Dct*-positive cells in control (Fig 4E and 4I), *Tfap2a* SCM (Fig 4F and 4J), and *Tfap2b* SCM (Fig 4G and 4K) embryos, but far fewer cells in *Tfap2a/Tfap2b* DCM embryos (Fig 4H and 4L). The absence of *Pmel*-positive and *Dct*-positive melanoblasts in DCMs was evident from the time these cells emerged in control embryos at E10.5 and E11.5, suggesting that the reduced melanoblast number in DCMs is not the result of impaired melanoblast migration (S8E–S8L Fig). Because TFAP2 paralogs have been shown to function during the early stages of neural crest induction [33,34], we next tested whether the observed reduction in melanocytes could be explained by a disruption in this step. Both lineage tracing with the *r26r*-reporter line (S9A–S9F Fig) and *in situ* hybridization with a *Sox10* riboprobe at E9.5 (S9G and S9H Fig) revealed relatively normal neural crest induction in DCMs, as in controls. Consistent with this observation, α-neurofilament immunostaining (Fig 4M and 4N) and lineage tracing (Fig 4O and 4P, S10A–S10D Fig) identified the initial formation of an alternate trunk neural crest derivative, dorsal root ganglia (DRG), in both DCMs and controls. However, similar to the melanocyte lineage, the neural crest-derived enteric nervous system (ENS) was disrupted in *Tfap2a* SCM embryos and completely failed to populate the gastrointestinal tract of DCM embryos (S10E–S10L Fig). Broadly, these analyses suggest that in mouse embryos, paralogous proteins TFAP2A and TFAP2B act redundantly subsequent to neural crest induction within distinct neural crest lineages. The virtual absence of melanoblasts in *Tfap2a/Tfap2b* DCM embryos implies that they contribute to specification and differentiation of the melanocyte lineage, similar to the functions of MITF.

### Zebrafish *mitfa;tfap2a* double mutants display a synergistic genetic interaction

**Fig 5:**
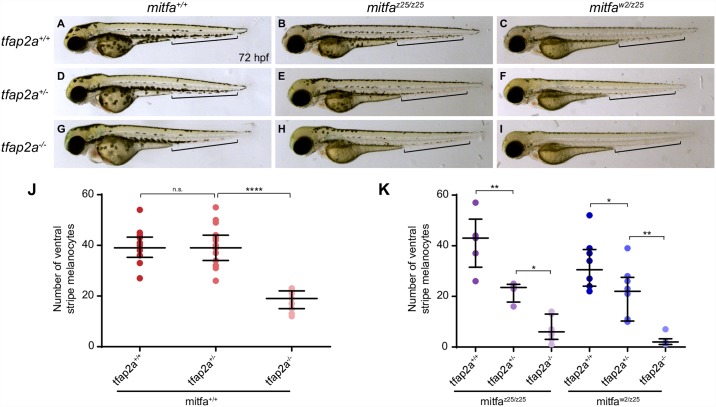
*tfap2a* and *mitfa* display a genetic interaction in zebrafish. (A-I) Compared to wildtype embryos at 72 hpf (A), melanocytes in *mitfa*^*z25/z25*^ mutant embryos (B) are dark but punctate, and melanocytes in *mitfa*^*w2/z25*^ trans-heterozygous embryos (C) are pale, punctate, and reduced in number. While heterozygous mutation of *tfap2a* in the wildtype background (*tfap2a*^*+/-*^) (D) does not result in a melanocyte phenotype, *mitfa*^*z25/z25*^*;tfap2a*^*+/-*^ mutants (E) and *mitfa*^*w2/z25*^*;tfap2a*^*+/-*^ mutants (F) display a loss of melanocytes in the ventral stripe (brackets). Similarly, the well-characterized phenotype of fewer, paler melanocytes in *tfap2a*^*-/-*^ null mutants (G) is more pronounced in both the *mitfa*^*z25/z25*^ mutant background (H) and the *mitfa*^*w2/z25*^ mutant background (I). (J, K) Counts of ventral stripe melanocytes (see brackets in A-I) showed no difference between wildtype and *tfap2a*^*+/-*^ embryos (J, n = 10+), while *tfap2a*^*+/-*^ embryos in both *mitfa* mutant backgrounds had significantly fewer melanocytes in the ventral stripe (K, n = 4–8). One-way ANOVA: *p<0.05, **p<0.01, ****p<0.0001.

Given the widespread co-occupancy of regulatory elements by TFAP2A and MITF, we predicted that *mitfa;tfap2a* double mutant zebrafish would have a greater-than-additive melanocyte phenotype consistent with synergistic interaction between these genes. To test this, we used the hypomorphic allele *mitfa*^z25^ to reduce Mitfa levels without eliminating the melanocyte lineage, as occurs with total loss-of-function alleles such as *mitfa*^*w2*^ [94,95]. Compared to wildtype embryos at 72 hpf (Fig 5A), *mitfa*^z25/z25^ homozygotes have fewer and less dendritic melanocytes (Fig 5B). Melanocytes are further reduced, punctate, and noticeably paler in *mitfa*^w2/z25^ trans-heterozygous embryos (Fig 5C). Incrosses of *mitfa*^*w2/z25*^;*tfap2a*^*+/-*^ double heterozygous adults and *mitfa*^*+/+*^*;tfap2a*^*+/-*^ single heterozygous adults yielded various combinations of *mitfa;tfap2a* genotypes. Embryos were sorted into phenotypic bins at 72 hpf, photographed individually, and genotyped for both *mitfa*^*z25*^ (when appropriate) and *tfap2a*. Relative to wildtype embryos (Fig 5A), *mitfa*^*+/+*^*;tfap2a*^*-/-*^ homozygous mutants exhibited a clear reduction of melanocytes in the ventral stripe caudal to the tail, reflecting decreased melanocyte migration and/or proliferation (Fig 5G). However, *mitfa*^*+/+*^*;tfap2a*^*+/-*^ heterozygous mutants could not be distinguished from wildtypes (compare Fig 5A and 5D). Quantification of ventral tail melanocytes (count area indicated by brackets in Fig 5A–5I) revealed that there is some variation within each genotype, but we detected no genotype/phenotype correlation between *tfap2a*^*+/+*^ and *tfap2a*^*+/-*^ embryos (Fig 5J, one-way ANOVA). In contrast, both the *mitfa*^z25/z25^ (Fig 5B, 5E and 5H) and *mitfa*^w2/z25^ (Fig 5C, 5F and 5I) backgrounds had three distinguishable phenotypic groups corresponding to the *tfap2a* genotype, with *tfap2a*^*+/-*^ embryos showing significant differences in the number of ventral tail melanocytes compared to both *tfap2a*^*+/+*^ and *tfap2a*^*-/-*^ embryos (Fig 5K, one-way ANOVA). Thus, the phenotype of double mutants appears to be more severe than the combination of the phenotypes in single mutants. The biochemical underpinning of this genetic interaction is unknown; Mitfa and Tfap2a may interact synergistically at one or more regulatory elements, or Mitfa may regulate *tfap2a* expression in melanocytes.

### Forced expression of *tfap2a* does not rescue melanocytes in *mitfa* mutant zebrafish

**Fig 6:**
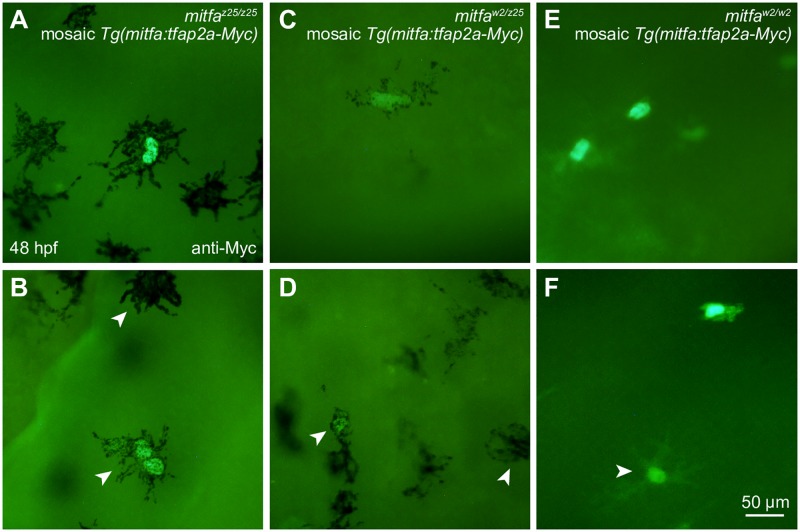
Forced expression of *tfap2a* does not rescue melanocytes in *mitfa* mutant zebrafish. (A-D) Nuclei of melanocytes expressing mosaic *Tg(mitfa*:*tfap2a-Myc)* are brightly labeled after anti-Myc immunostaining. At 48 hpf, labeled melanocytes in *mitfa*^*z25/z25*^ mutant embryos (A, B) and *mitfa*^*w2/z25*^ mutant embryos (C, D) display no apparent improvement in pigmentation or dendricity compared to adjacent unlabeled cells (B and D, white arrowheads). (E, F) In *mitfa*^*w2/w2*^ mutants, all labeled cells are unpigmented and resemble other cell types including xanthophores (F, white arrowhead).

Genetic interaction between *tfap2a* and *mitfa* supports the idea that the factors encoded by these genes regulate shared targets in melanocytes, possibly within single or converging pathways. Previously, we found that elevating expression of *mitfa* (under the *sox10* promoter) partially compensates for the reduction of Tfap2 in *tfap2a/tfap2e*-depleted zebrafish embryos [30]. Thus, we next investigated whether the reciprocal experiment, artificially elevating expression of *tfap2a*, could rescue melanocyte development in the absence or reduction of Mitfa. To test this, we engineered the *tfap2a* cDNA to encode 6 Myc epitopes at the carboxy terminus. To confirm that the epitope-tagged Tfap2a was still functional, we injected *tfap2a-Myc* mRNA into embryos depleted of *tfap2a* and *tfap2c* and observed differentiated melanocytes, which are otherwise completely absent from such animals [33]. We then fused *tfap2a-My*c cDNA downstream of the *mitfa* promoter, which is active in melanocytes (*mitfa* promoter described in [96]), and injected the *mitfa*:*tfap2a-Myc* plasmid into embryos derived from an incross of *mitfa*^*w2/z25*^ adults. At 48 hpf, embryos were sorted into three groups based on the *mitfa* mutant phenotype, fixed, and subsequently processed for anti-Myc immunoreactivity. Ten plasmid-injected embryos and ten uninjected control embryos were documented in each group. We detected brightly-labeled cells, including several melanocytes, in both *mitfa*^*z25/z25*^ (Fig 6A and 6B) and *mitfa*^*w2/z25*^ (Fig 6C and 6D) plasmid-injected embryos, whereas uninjected controls had no labeled cells. However, the anti-Myc immunoreactive melanocytes were phenotypically indistinguishable from neighboring unlabeled melanocytes by morphology, dendricity, or pigmentation (Fig 6B and 6D, arrows). The *mitfa*^*w2/w2*^ plasmid-injected embryos had similar numbers of anti-Myc immunoreactive cells to the other genotypes, but none showed any hint of pigmentation (Fig 6E and 6F). We did observe a highly dendritic but wholly unmelanized cell, which is likely to be a xanthophore (Fig 6F, arrow). In summary, elevating *tfap2a* expression under the *mitfa* promoter was not sufficient to rescue melanocytes in *mitfa*^*w2/w2*^ null mutants, nor did it improve the quality or pigmentation of melanocytes in *mitfa*^*z25/z25*^ and *mitfa*^*w2/z25*^ mutants. We conclude that, despite regulating many of the same targets, Tfap2a is unable to replace Mitfa in the melanocyte lineage, at least at the dose of over-expression tested here.

## Discussion

The current work presents an explanation for the basis of pigmentation phenotypes in zebrafish and mouse *Tfap2a* mutants, suggests that redundant activity of TFAP2 paralogs is responsible for the relative mildness of these phenotypes, and reveals a previously unappreciated role for TFAP2 alongside MITF in melanocyte differentiation. Microarray analysis on the trunks of zebrafish *tfap2a*^*-/-*^ mutants showed that several genes with Tfap2a-dependent expression *in vivo* are known to be important for melanization of embryonic melanophores (*oca2* [97], *tyr* [98], and *slc45a2/albino* [99]). The phenotype of delayed melanization in these mutants is plausibly explained by reduction of these genes, as well as others that may have escaped detection due to Tfap2a-dependent expression in melanocytes but not in other cell types (one example of this is *kita* [27,29]). Analysis of mouse melanocytes depleted of *Tfap2a* revealed an overlapping but shorter list of TFAP2A-dependent genes, potentially due to incomplete *Tfap2a* knockdown in these cells. Species dependent differences are also possible. For example, expression of *Dct*, but not of *Irf4* or *Tyr*, was TFAP2A-dependent in mouse melanocytes, while the opposite trend was seen in the human 501mel cell line [37]. TFAP2A ChIP-seq results from mouse and human melanocytes integrated with H3K27ac ChIP-seq, marking active regulatory elements, revealed that the majority of TFAP2A-dependent pigmentation genes were direct transcriptional targets of TFAP2A. Indeed, far more genes, including most of those mutated in mice with coat color phenotypes, were associated with TFAP2A-bound active regulatory elements than were found to have TFAP2A-dependent expression (at the level of detection of our assays). Three results indicate that compensation by other TFAP2 paralogs is the most likely explanation for why more genes do not appear to be TFAP2A-dependent. First, deletion of TFAP2 binding sites reduced the promoter activity of a gene that was relatively unaffected by loss of TFAP2A in the expression analyses. Second, depletion of *tfap2e* in zebrafish delays melanocyte differentiation, but only in the context of *tfap2a*^-/-^ mutants [30], suggesting that these paralogs are at least partially redundant in function. Third, we find here that in the mouse embryo, neural crest-specific deletion of either *Tfap2a* or *Tfap2b* alone does not greatly impact embryonic development of the melanocyte lineages, but the combined knockout of both genes causes a significant loss of melanocytes. Thus, TFAP2 paralogs promote induction of the neural crest lineage and subsequently promote differentiation of one of its derivatives; they have a similar feed-forward quality in epidermis [100]. It is important to note the possibility that TFAP2 paralogs act throughout the specification, proliferation, and differentiation of both neural crest and melanocyte lineages such that disruption at any of these steps ultimately results in a melanocyte phenotype. For example, almost complete loss of melanoblasts in *Tfap2a/Tfap2b* DCM mice could reflect a requirement for TFAP2 in neural crest survival or lineage specification of certain derivatives. Similarly, the genetic interaction between *mitfa* and *tfap2a* in zebrafish appears to be primarily due to reduced cell number, as well as defects in melanoblast migration. While further study will be required to uncouple these various functions of TFAP2 paralogs at each step of melanocyte development, the results described here strongly support a role for TFAP2A in the terminal differentiation of melanocytes. Overall, our findings suggest that TFAP2A, acting in partial redundancy with other TFAP2 paralogs, joins MITF, SOX10, YY1, LEF1, and IRF4 in directly regulating the expression of melanocyte differentiation effector genes [20,37,101].

We observed widespread co-occupancy of TFAP2A and MITF at active regulatory elements, but it is unknown whether the two transcription factors bind such elements cooperatively. In support of this possibility, we observed a genetic interaction between *mitfa* and *tfap2a* affecting melanocyte development in zebrafish. However, an interaction would also be expected if Tfap2a and Mitfa act in a single pathway. Although *mitfa* expression in melanocytes is not strongly Tfap2a-dependent, it is possible that expression of *tfap2a* in melanocytes is Mitfa-dependent, as levels of TFAP2A protein were reduced in 501mel cells depleted of *MITF* with an shRNA [37]. In a published mass-spectrometric analysis of proteins that immunoprecipitate with MITF, TFAP2A peptides were not identified [44]. However, another similar experiment did identify low levels of TFAP2A peptides, although TFAP2A did not detectably co-immunoprecipitate with an epitope-tagged MITF (J. P. Lambert, A. C. Gingras, personal communication). The strength and importance of any physical interaction among TFAP2A, MITF, SOX10, and other transcription factors bound at regulatory elements active in melanocytes requires further investigation. There is evidence that TFAP2A serves as a pioneer transcription factor for androgen receptor in epididymis cells [68], and as TFAP2A expression precedes MITF expression in the melanocyte lineage, it is conceivable that TFAP2A plays a similar role for MITF. Conversely, the inability of Tfap2a to substitute for loss of Mitfa in zebrafish, at least at the doses tested here, is consistent with MITF serving as a pioneer factor for TFAP2A. It is also possible that TFAP2A and MITF bind independently, but nonetheless have a cooperative effect on gene expression, as indicated by *in vitro* tests of an intronic enhancer of the *IRF4* gene [37]. Analysis of TFAP2A chromatin binding in cells depleted of MITF, and of MITF chromatin binding in cells depleted of relevant TFAP2 paralogs, might address whether either protein is required to make the chromatin accessible for the other.

In metastatic melanoma, the levels of MITF activity have been proposed to control the cellular phenotype: high levels promote melanocyte proliferation and differentiation, while lower levels confer an invasive state [22,102]. It is notable that TFAP2A levels are decreased in advanced-stage melanoma tumors versus earlier stage melanoma and nevi, whereas MITF expression levels are relatively constant (data from The Cancer Genome Atlas [46]). Furthermore, elevating levels of TFAP2A in A375SM cells was reported to inhibit tumorigenicity and metastatic potential in nude mice [103]. Based on this evidence, it is possible that the level of MITF activity is adjusted through loss or gain of an essential collaborator, TFAP2A (and perhaps its paralogs). Here, we find that TFAP2A peaks are associated with many genes encoding regulators not only of melanocyte differentiation, but also of other cellular phenotypes purported to be governed by MITF, such as growth and senescence. The melanoma subtype that is most difficult to target therapeutically, and which is thought to depend on a relatively low level of MITF activity, has stem cell qualities, an invasive phenotype, and an expression profile resembling that of neural crest [104]. Thus, early requirements for Tfap2a and its paralog Tfap2c observed in zebrafish neural crest induction [33,34] may suggest a role for TFAP2 in this invasive subtype of melanoma as well. Further investigation will be necessary to determine the potential tumor-promoting or tumor-inhibiting consequences of TFAP2A expression (or activity) levels in melanoma.

## Materials and methods

### Cell culture

#### Mouse melan-a cells

Mouse immortalized melanocytes, Ink4a-ARF double null melan-a cells, were maintained as described previously [105].

#### Human primary melanocytes

Primary melanocytes were regularly maintained in culture as described previously [106], using complete growth medium consisting of Minimum Essential Medium Eagle (MCDB) 153 supplemented with 4% fetal bovine serum, insulin (5 μg/ml), α-tocopherol (1 μg/ml), 1% penicillin/streptomycin/amphotericin, human basic fibroblast growth factor (bFGF, 0.6 ng/ml), phorbol 12-myristate 13-acetate (PMA, 8 nM), and bovine pituitary extract (BPE, 13 μg/ml). Primary melanocytes were grown in T-75 cm^2^ flasks. To split flasks, cells were washed for 2 min in EDTA solution and trypsinized for 2 min in 500 μl of a 0.25% trypsin solution.

#### Human melanoma cells

The M21 melanoma cell line was obtained from the American Type Culture Collection (ATCC, Manassas, VA), and maintained in RPMI containing 10% FBS and 1% PenStrep.

### Microarrays and qRT-PCR

#### Zebrafish

*tfap2a*^*-/-*^ mutant [lockjaw allele, 28] and non-mutant siblings (i.e. wildtype and *tfap2a*^*+/-*^ heterozygous) zebrafish larvae were sorted at 36 hpf based on pigmentation phenotype, and heads were removed to eliminate the RPE. Pools of 25–30 embryos were collected in TRIzol (Invitrogen), and RNA was extracted according to the manufacturer protocol. RNA was further purified using the RNeasy Mini Kit (Qiagen) and tested for quality using an Experion bioanalyzer (Bio-Rad). The SuperScript Double-Stranded cDNA Synthesis Kit (Invitrogen) was used to generate ds-cDNA, which was also tested for quality on the Experion bioanalyzer. ds-cDNA was labeled using Cy3-coupled random nanomers (Nimblegen). Biological replicates were hybridized to sub-arrays of Roche Nimblegen gene expression microarrays (12 x 135K, design number 090505_Zv7_EXPR). The arrays were scanned on an Axon GenePix 4200A microarray scanner (Molecular Devices). Raw data (Pair files) were normalized in ArrayStar software, version 12.0.0 (DNASTAR, Inc). Background correction, quantile normalization, and median polish summarization were performed by applying the robust multichip analysis (RMA) algorithm. A Student’s *t*-test, corrected for multiple testing by the Benjamini and Hochberg false discovery rate (FDR) method, was performed for each pair-wise comparison. Transcripts were considered differentially expressed if the FDR-adjusted P-value was less than 0.05. Primers for qRT-PCR were designed to amplify fragments of 150–200 bp spanning exon junctions (listed in S11 Table). qRT-PCR was carried out using iQ SYBR Green Supermix (Bio-Rad) on a CFX96 Real-Time PCR Detection system (Bio-Rad) according to the default settings. Three technical replicates were averaged for each sample, and the 2^-ΔΔCt^ method was used to calculate the difference in gene expression between samples, after normalization to the reference gene *b2m*. Statistical significance was evaluated via a Mann-Whitney U test for each gene.

#### Mouse

Melan-a cells were transfected in triplicate with either of two independent siTfap2a double strand duplex RNAs, siA = sure select siRNA #s74846 (Ambion), si5 = MMS.RNAIN011547.12.5 (Integrated DNA Technologies), or a non-silencing control siRNA NC-1 (Integrated DNA Technologies) using Lipofectamine RNAimax protocol (Invitrogen) with 25pmol of oligo per 6 well dish. Total RNA isolated from each bioreplicate was used to synthesize cDNA for hybridization to Mouse Gene 2.0 ST Arrays (Affymetrix). cDNA from each bioreplicate was then assayed by qRT-PCR with 3X technical replicates using TAQMAN qPCR probes (ABI, listed in S11 Table), on a StepOne machine (Thermo Fisher) with standard fast protocol conditions. Relative sample quantifications were performed using a standard curve method, where gene expression for each bioreplicate was normalized to *Actb*, then plotted relative to non-silencing control samples. Statistical significance was evaluated via one-way ANOVA with Bonferroni multiple test correction for each gene when response to both siA and si5 agreed.

### ChIP-seq and ChIP-qPCR

#### Mouse

Melan-a cell ChIP-seq experiments (2 replicates) were performed as described by Gorkin and colleagues with the following alterations [65]: sonication was performed using a Q500 horn sonicator (Qsonica), 70% amplitude, 25 minute sonication cycle occurring with a 10 second pulse, 20 second off setting. Peaks that overlap by at least 1 bp between replicates were used for subsequent analyses. Anti-TFAP2A and IgG ChIP samples from mouse melan-a cells were validated by ChIP-qPCR with primer sets listed in S11 Table. Quantification of PCR products was performed as per manufacturer’s instructions using FAST SYBR Green master mix (ABI) and the Step-One Plus Real-time PCR machine (ABI) under FAST standard cycling conditions.

#### Human

ChIP was performed on human primary melanocytes that had not exceeded 10 passages using 10 μg anti-TFAP2A (3B5, Santa Cruz Biotechnology, Inc.) or control Ab-108C polyclonal Goat IgG control (R&D Systems). Over 45 million mapped reads were obtained for one biological replicate and one input control sample. Approximately 40 million human primary melanocytes were fixed at room temperature for 10 minutes with 1% formaldehyde diluted in cell culture media (v/v). Following fixation, formaldehyde was quenched with 1.25M glycine for 5 minutes at room temperature. Fixed/quenched cells were subsequently spun at 1,200 rpm for 5 minutes, washed with 1x PBS, and respun. Pelleted cells were then resuspended in 1mL cell lysis buffer (150mM NaCl, 10mM Hepes pH 7.4, 1.5mM MgCl2, 10 mM KCl, 0.5% NP-40, 0.5mM DTT, 1mM EDTA, plus protease inhibitors) and incubated on ice 10 minutes. Following cell lysis, nuclei were pelleted by spinning at 5,000 rpm, for 5 minutes at 4°C. Nuclei were then resuspended in 1 part nuclear lysis buffer (50mM Tris pH 8.0, 10mM EDTA, 1% SDS plus protease inhibitors) and 2 parts ChIP-dilution buffer (150mM NaCl, 16.7mM Tris pH 7.5, 3.3mM EDTA, 1% Triton X-100, 0.1% SDS, 0.5% Na-Doc, plus protease inhibitors), and sonicated using either a Covaris instrument (Model S220) or with a standard probe-tip sonicator (VirTis Virsonic 600). Sonication settings were determined empirically to generate fragments ranging from 200–500 bp. Following sonication samples were spun at 14,000 rpm for 15 minutes, and supernatant was saved for immunoprecipitation. For immunoprecipitation, Dynabeads (Life Technologies) were first washed 3x with ChIP-dilution buffer and subsequently incubated with equal amounts (5–10ug) of either anti-TFAP2A (3B5, Santa Cruz Biotechnology, Inc.) or Mouse IgG (Millipore) for 2 hours to overnight at 4°C. Following incubation, antibody-bound beads were washed 3x with ChIP-dilution buffer. Sonicated chromatin was equally split between two tubes, brought to approximately 1 mL with ChIP-dilution buffer, and used to resuspend antibody bound beads. Samples were incubated at 4°C overnight. Antibody/bead/chromatin complexes were then washed with a series of solutions 3x and eluted with 100 μL elution buffer. Crosslinks were reversed overnight at 65°C with 0.2 M NaCl. DNA was further cleaned using a Qiagen PCR purification kit as per manufacturer’s instructions (Qiagen). Total enriched DNA was quantified using a PicoGreen dsDNA assay kit (Life Technologies) and shearing efficiency was confirmed using a High Sensitivity DNA Analysis Kit and a 2100 Bioanalyzer (Agilent Technologies) on the input sample. Libraries were then constructed using ABI's SOLiD ChIP-seq kit with barcoding (rev. 08/06/2010), as per manufacturer’s instructions (Life Technologies). Following library construction, samples were sequenced using the ABI SOLiD 3.0 platform, using a 50bp sequencing run. LifeScope Genomic Analysis Software was used for basecalling and alignment to hg19 genome with default specifications for each program. Quality assessment was performed using Phantompeak tools as per the guidelines of the Encode project. Peaks were called using the SPP R program with an FDR setting of 0.05.

ChIP-qPCR was performed to validate chromatin enrichment at known TFAP2A targets using primer sets listed in S11 Table. Quantification of PCR products was performed as per manufacturer’s instructions using iQ SYBR Green Supermix (Bio-Rad) on a CFX96 Real-Time PCR Detection system (Bio-Rad) according to the default settings. Three technical replicates were averaged for each sample, and the 2^-ΔΔCt^ method was used to calculate the differences in gene expression.

### ChIP-seq data analysis

The ChIP-seq Tool Set in Galaxy was used for all peak overlap analyses [107]. For comparison of human ChIP-seq peaks to gene expression, we used a published RNA-seq expression profile of human penis foreskin melanocytes from the Roadmap Epigenomics Project (GEO accession number, GSM958174) [64]. Motif enrichment analysis was carried out using the MEME-ChIP suite [108], including CentriMo [60], MEME-ChIP [69], and AME [109] tools. For gene set enrichment analysis, we used the Genomic Regions Enrichment of Annotations Tool (GREAT), with the association rule basal plus extension, proximal: 5 kb upstream, 1 kb downstream, plus distal: up to 100 kb [72]. ChIP-seq read density clustering analysis and quantitative comparisons were performed using *k*-means linear enrichment cluster function in seqMINER with the following parameters: window size = -5K to +5K, read extension = 200bp, seed = 12 [110] (http://bips.u-strasbg.fr/). The Panther Classification System was used for GO term enrichment analysis on gene lists [76,77].

#### Super-enhancer analysis

Human foreskin melanocyte H3K27ac ChIP-seq alignment results were obtained from the Roadmap Epigenomics Project (GEO accession number, GSM1127072) [64]. Typical enhancers and super-enhancers were called using HOMER [111]. Briefly, all the stitched enhancer peaks were generated and sorted based on normalized tag count in descending order. Super-enhancers were defined by slope>1 (slope = Δ(normalized tag count) / Δrank) [84]. All stitched enhancers were compared with human melanocyte TFAP2A ChIP-seq peaks and MITF ChIP-seq peaks [18] using BEDTools (v. 2.24.0) [112]. We then normalized the rank by the total number of stitched enhancers and plotted this against normalized super-enhancer score (obtained by calculating normalized tag counts / highest normalized tag count).

### RNA-seq in mouse melan-a cells

RNA sequencing (RNA-seq) libraries were prepared with the TrueSeq stranded mRNA kit (Illumina) and sequenced on the Illumina HiSeq 2000 platform. The ten 5’-most bases were trimmed from all of the raw RNA-seq reads. Reads were aligned to the mouse reference genome sequence (mm9) using the STAR alignment software (v. 2.3.0e). RNA-seq reads derived from rRNAs were removed using the split_bam.py script available in RSeQC (v. 2.3.7), using genomic locations of known rDNAs that were downloaded from UCSC. Counts for RNA-seq reads mapping to Ensembl-annotated transcripts (release 67) were calculated using the htseq-count software (v. 0.5.3p3). These raw RNA-seq counts were used for differential gene expression analysis that was performed using DESeq2 (v. 1.10.1).

### Promoter deletion analysis

The following versions of the *TRPM1* promoter sequence (748 bp) were obtained as double stranded gBlocks gene fragments from IDT: intact sequence, ΔAP2A with mutations in four TFAP2A binding sites, and ΔE1 with a mutation in the main MITF binding site. The consensus TFAP2A binding site GCCNNNGG was disrupted by changing the two underlined bases to T, whereas the E1 MITF site was changed as previously published by [86]. Fragments were cloned into a Tol2-cfos-FFluc vector via Gibson assembly and confirmed with Sanger sequencing.

M21 melanoma cells were grown to 70–90% confluency in a 24-well culture plate. In each well, the reporter plasmid (1μg) and a β-galactosidase plasmid (100ng) were transfected using Lipofectamine 3000 (Life Technologies). Approximately 48 hours after transfection, luciferase assays were conducted using the Dual-Light System from Applied Biosystems, and 20/20n Luminometer (Turner Biosystems, Sunnyvale, CA) according to the manufacturer protocols. Briefly, cells were washed with cold PBS and incubated in 40 μL lysis solution on a shaker for 15 minutes. Cell lysates were transferred to tubes and centrifuged at 12000 rpm for 2 minutes at 4°C, and supernatant was transferred to a clean tube. 10 μL of cell lysate was added to 25 μL of Buffer A and placed in the luminometer, where the injector adds 100 μL Buffer B/Galacton-Plus substrate and reads the signal after 1 second. Samples were incubated in the dark for 30 minutes before injection of 100 μL Accelerator-II and measurement of the β-gal signal after 1 second. For each version of the *TRPM1* promoter element, transfection was carried out in triplicate. Firefly luciferase reads from each sample were normalized to the respective β-gal reads, and an average signal was calculated within groups.

### Mouse genetics and immunostaining

Experiments utilizing mice in this study were carried out in strict accordance with the recommendations in the Guide for the Care and Use of Laboratory Animals of the National Institutes of Health. The protocol was approved by the Institutional Animal Care and Use Committee of the University of Colorado Denver. Noon on the day a copulatory plug was present was denoted as embryonic day 0.5. Mice used in this study included males that were heterozygous for a *Tfap2a*-null allele [113], heterozygous for a newly generated *Tfap2b*-null allele (EVO/TW, in preparation), and hemizygous for the *Wnt1-Cre* transgene [89] (*Tfap2a*^*null/wt*^*;Tfap2b*^*null/wt*^*;Wnt1-Cre*). These males were crossed with females that were homozygous for both a *Tfap2a*-conditional [24] and *Tfap2b*-conditional (EVO/TW, in preparation) allele, resulting in a 1:8 frequency of generating single or double conditional mutants, as well as various other genotype combinations. Mice were maintained on an outbred Black Swiss background. Of note, by virtue of this breeding scheme, the single conditional mutants would always be conditionally heterozygous for the alternate paralog. For *r26r* [90] and ‘tomato’ [114] experiments, the female was also homozygous for the reporter allele. Yolk sacs or tail clips were used for genotyping. DNA for PCR was extracted using DirectPCR Lysis Reagent (Viagen Biotech. Inc) plus 10 ug/ml Proteinase K (Roche) followed by heat inactivation at 85°C for 45 min. Samples were then used directly for PCR-based genotyping using allele specific primers (available upon request) at a concentration of 200 nM using the Qiagen DNA polymerase kit, including the optional Q Buffer solution (Qiagen).

With the exception of whole-mount β-galactosidase (β-gal) staining, procedures used for mouse embryo analysis (*in situ* hybridization, ɑ-neurofilament immunostaining, and immunofluorescence) have all been previously described [115]. Following euthanasia, embryos were collected at the indicated time-points in DEPC-PBS and subsequently processed. Briefly, for *in situ* hybridization, after embryo collection, trunks were removed, fixed overnight in 4% paraformaldehyde (PFA), and stained using the indicated riboprobe (*Pmel*, *Dct*, *Sox10*). For immunostaining, embryos were processed using an ɑ-neurofilament primary antibody [116] (IgG clone 2H3, obtained from the Developmental Studies Hybridoma Bank–University of Iowa), followed by colorimetric staining using a standard secondary antibody and DAB-detection. For β-gal immunofluorescence, collected embryos underwent a short fixation in 0.25% glutaraldehyde, were taken through a series of sucrose/O.C.T. solutions (Tissue-Tek O.C.T. Compound, Electron Microscopy Sciences), until being embedded in 100% O.C.T. and frozen on dry-ice. Cryosections were then cut at ~12μM on a Leica CM 1900 cryostat (Leica Biosystems Inc.) using the hindlimb as a delimiting rostral-caudal boundary between samples. Following sectioning, slides were washed in PBS, 2 x 10 min, blocked in 3% BSA (in PBS) 1 hr at room temperature, incubated over-night with a rabbit polyclonal anti-β-gal antibody (Product 55976, MP Biomedicals, LLC) diluted 1:200 in block solution. Subsequently, sections were washed 2 x 10 min in PBS, followed by a 1 hr incubation in goat-anti-rabbit Alexa Fluor 488 (Thermo Fisher) and counterstained with DRAQ5 (Abcam), washed again 2 x 10 min in PBS, and then cover-slipped. Processed samples were imaged on a Leica TCS SP5 II confocal microscope and representative images taken. Finally, for β-gal staining, embryos were collected at appropriate time points and fixed ~1hr at room temperature with 0.25% glutaraldehyde in PBS. Subsequently, embryos were washed 3 x 30 minutes in a ‘lacZ rinse buffer’ (0.2M sodium phosphate, 2mM magnesium chloride, 0.02% NP40, and 0.01% sodium deoxycholate), and then incubated overnight in a ‘lacZ staining solution’ (lacZ rinse buffer plus 5mM potassium ferricyanide, 5mM potassium ferrocyanide, and 1 mg/ml X-gal) at 37°C. Following adequate staining, embryos were post-fixed in 4% PFA overnight, moved to PBS, and subsequently imaged.

### Zebrafish genetics and immunostaining

Zebrafish alleles used in this study include *tfap2a*^*low*^ [lockjaw allele, 28], *mitfa*^*w2*^ [94], and *mitfa*^*z25*^ [95]. Lines were maintained as *mitfa* trans-heterozygous and *tfap2a* heterozygous (*mitfa*^*w2/z25*^*;tfap2a*^*+/-*^) or *mitfa* wildtype and *tfap2a* heterozygous (*tfap2a*^+/-^). For genetic interaction experiments, *mitfa*^*w2/z25*^*;tfap2a*^*+/-*^ animals were incrossed to generate *tfap2a*^*+/+*^, *tfap2a*^*+/-*^, and *tfap2a*^*-/-*^ genotypes in each of *mitfa*^*z25/z25*^ and *mitfa*^*w2/z25*^ backgrounds. To obtain control animals in the *mitfa*^*+/+*^ background, *mitfa*^*+/+*^*;tfap2a*^*+/-*^ animals were also incrossed. At 72 hpf, embryos were binned according to melanocyte phenotype, photographed, and genotyped via High Resolution Melt Analysis (HRMA) using Precision Melt Supermix (Bio-Rad) on a CFX96 Real-Time PCR Detection system (Bio-Rad) according to the default settings. Precision Melt Analysis Software (Bio-Rad) was used to analyze melt curves. HRMA primer sequences are as follows: *tfap2a*^*low*^ (forward: GTA GCT ATG TTT CGT GGT TA; reverse: ACA ATA AGC AGC TGC TTT AC), *mitfa*^*z25*^ (forward: GCA GAA GTC AGA GCC CTG GC; reverse: ACG GAT CAT TTG ACT TGG GAA TTA AAG). Ventral stripe melanocytes were counted from lateral-view images taken at 72 hpf and statistical significance between groups was tested using a one-way ANOVA with multiple test correction.

For zebrafish rescue experiments, *mitfa*^*w2/z25*^ trans-heterozygotes were incrossed to obtain *mitfa*^*z25/z25*^, *mitfa*^*w2/z25*^, and *mitfa*^*w2/w2*^ genotypes, which were clearly distinguishable based on melanocyte phenotype at 48 hpf. 3-way Gateway cloning technology was used to generate a pDestTol2CG2 plasmid [117] containing 5’ entry *mitfa* promoter [96], middle entry *tfap2a* cDNA, and 3’ entry 6x Myc epitope tag followed by polyA, resulting in the presence of Myc epitope tags on the carboxy terminus of Tfap2a. This construct was then injected into the above cross along with Tol2 mRNA, both at a concentration of 30 ng/uL. Due to the presence of a *cmlc2*:*GFP* reporter in the vector, injected embryos were screened for successful plasmid integration based on expression of GFP in the heart at 28–30 hpf. GFP positive embryos and uninjected control embryos were fixed overnight in 4% paraformaldehyde at 48 hpf. After fixation, embryos were rinsed 3x in PBS, blocked in PBDT+2.5% goat serum for 1 hr at room temperature, and incubated in anti-Myc primary overnight at 4°C (9E10, obtained from the Developmental Studies Hybridoma Bank—University of Iowa, 1:100 diluted in block solution). The following day, embryos were rinsed 4x in PBS + 0.1% Triton X-100 (PBS-Tx) and incubated overnight at 4°C in Alexa Fluor goat-anti-mouse 488 secondary (Thermo Fisher) diluted in block solution. Embryos were then rinsed 4x15 minutes in PBS-Tx and mounted on slides. Ten individual embryos of each genotype with plasmid injection or uninjected control were viewed at 40x and photographed.

### Immunostaining in cell lines

Cells were grown to approximately 75% confluency in 24-well plate on poly-lysine coated disks and fixed with 4% paraformaldehyde for 1 hr at 4°C. Following fixation, cells were rinsed 3x20 minutes with 1x PBS, permeabilized in 1x PBS + 0.3% Triton X-100 for 30 min at 37°C, rinsed 3x10 minutes in PBS-Tx, and blocked (1% BSA in PBS-Tx) overnight at 4°C. Cells were then incubated in anti-TFAP2A primary overnight at 4°C (3B5, Santa Cruz Biotechnology, Inc., 1:100, 250ug/mL stock diluted in block solution). The following day, cells were rinsed 3x20 minutes in PBS-Tx and incubated for 2 hr at room temperature in Alexa Fluor goat-anti-mouse 488 secondary (Thermo Fisher) diluted in block solution. Cells were then rinsed 4x15 minutes in PBS-Tx and disks mounted in Prolong Gold antifade reagent and DAPI SlowFade solution (Life Technologies). Slides were imaged on a Zeiss LSM 700 Flexible Confocal Microscope (Carl Zeiss Microscopy). Briefly, for quantification of immunofluorescent intensity approximately three 20x images were taken of each slide, and three slides of each cell line processed (including a no primary control). Each image included both the antibody of interest as well as DAPI staining to identify cell nuclei.

### Ethics statement

All experiments were approved by the University of Iowa or University of Colorado Institutional Animal Care and Use Committee (IACUC). We abide by PHS Policy, USDA-Animal Welfare regulations, the Guide for the Care and Use of Laboratory Animals, University of Iowa policies and regulations and any state and local laws and regulations.

## Supporting information

S1 Fig Time course of delayed melanocyte differentiation in *tfap2a*^*-/-*^ zebrafish. (A-L) Lateral views of live *tfap2a*^*+/-*^ (left column) and *tfap2a*^*-/-*^ (right column) zebrafish embryos at the indicated age from 28-38 hpf. (C, I) Melanocytes in a *tfap2a*^*+*/-^ zebrafish (C) appear darkly pigmented by 32 hpf, whereas melanocytes at the same location in a *tfap2a*^*-/-*^ mutant (I) at this stage remain pale and punctate. (F, L) At 38 hpf, there is still a detectable difference in the level of pigmentation in *tfap2a*^*+*/-^ (F) and *tfap2a*^*-/-*^ (L) mutant animals.
(PDF)

S2 Fig Nuclear anti-TFAP2A immunoreactivity is detected in primary melanocytes and M21 melanoma cells. (A-E) Nuclear anti-TFAP2A immunoreactivity (green fluorescence) in human primary melanocytes (A) and four human melanoma cell lines (B-E). Inset shows DAPI counter-stain for nuclei. TFAP2A expression is low but detectable in M21s (C).
(PDF)

S3 Fig TFAP2A ChIP-qPCR confirms peaks in three melanocyte or melanoma cell lines. (A, B) Validation of TFAP2A ChIP-seq in mouse melan-a cells. (A) CentriMo analysis shows that mouse TFAP2A ChIP-seq peaks are centered on the TFAP2A/TFAP2C binding motif (TFAP2A p = 1.8e-2332, TFAP2C p = 2.7e-2186) (B) Four of five sites tested by ChIP-qPCR confirmed enrichment of TFAP2A binding over the IgG control, whereas an off-target site (*Nos2* -50kb) showed no enrichment. (C, D) Validation of TFAP2A ChIP-seq in human primary melanocytes. (C) CentriMo analysis shows that human TFAP2A ChIP-seq peaks are centered on the TFAP2A/TFAP2C binding motif (TFAP2A p = 8.1e-820, TFAP2C p = 8.2e-785). (D) Five of six sites tested by ChIP-qPCR confirmed enrichment of TFAP2A binding over the IgG control, whereas an off-target site (chrm17) showed no enrichment. (E) Validation of TFAP2A ChIP-seq in M21 melanoma cells. Four of five sites tested by ChIP-qPCR confirmed enrichment of TFAP2A binding over the IgG control, whereas an off-target site (chrm17) showed no enrichment. Notably, *DCT* showed enrichment for TFAP2A binding in the mouse melan-a cells, but not in human primary melanocytes or M21s, which is consistent with the ChIP-seq results for mouse and human, respectively.
(PDF)

S4 Fig TFAP2A binds the promoters of genes that are highly expressed in human primary melanocytes. (A) Pie chart showing distribution of human TFAP2A peaks with respect to genomic features. TSS, transcription start site; TTS, transcription termination site. (B) Distance from TSS to the nearest TFAP2A peak for genes in three expression categories: highest 1000, median 1000, or lowest 1000. Highly expressed genes are more likely to have promoter-proximal TFAP2A peaks. Human expression data from the Roadmap Epigenomics Project GSM958174 [64].
(PDF)

S5 Fig TFAP2A peaks overlap active enhancer signatures near melanocyte genes in mouse melan-a cells. (A-F) UCSC genome browser tracks showing mouse TFAP2A ChIP-seq peaks that overlap an active enhancer signature (p300 flanked by H3K4me1) or a partial enhancer signature near the promoters of (A) *Pmel*, (B) *Irf4*, (C) *Mc1r*, (D) *Mitf*, (E) *Oca2*, and (F) *Tyr*.
(PDF)

S6 Fig Genes associated with TFAP2A peaks in human and mouse melanocytes are enriched for melanocyte-related GO terms. (A, B) GO term enrichment analysis of genes associated with (A) 13,690 TFAP2A peaks in human melanocytes and (B) 16,305 TFAP2A peaks in mouse melanocytes. Gene association was identified using the GREAT algorithm with an assignment rule of basal plus extension, proximal TSS -5/+1kb, distal up to 100kb. For each category, the top five most significant terms are listed, followed by select terms of interest. Significance is charted as -log10(binomial p-value). (C) Density-based clustering of H3K27ac signal at TFAP2A peaks in mouse melanocytes (H3K27ac data from [14]).
(PDF)

S7 Fig Analysis of H3K27ac signal identifies 652 super-enhancers in human primary melanocytes. Chart depicting typical enhancers (gray) and super-enhancers (black) in human melanocytes.
(PDF)

S8 Fig Time-series of melanocyte development in mouse control and *Tfap2a/Tfap2b* DCM embryos. (A-D) Dorsal views of E12.5 control (A), *Tfap2a* SCM (B), *Tfap2b* SCM (C), or *Tfap2a/Tfap2b* DCM (D) mouse embryos processed for β-galactosidase (β-gal) staining, labeling neural crest cells and their derivatives, including melanocytes migrating on the ventrolateral pathway (as in Fig 4A–4D). (E-L) Lateral views of E10.5 control (E, I) or *Tfap2a/Tfap2b* DCM (F, J) and E11.5 control (G, K) or *Tfap2a/Tfap2b* DCM (H, L) mouse embryos processed for *Pmel* (E-H) or *Dct* (I-L) expression by *in situ* hybridization. Yellow lines (in E, I, G, K) indicate extent of rostral-caudal regions of *in situ* signal, rostral left. Abbreviations: DCM, double conditional mutant; fl, forelimb; hl, hindlimb; SCM, single conditional mutant (A = *Tfap2a* or B = *Tfap2b*). Scale bars = 500μM.
(PDF)

S9 Fig Neural crest expression patterns appear normal at early stages in mouse *Tfap2a/Tfap2b* DCM embryos. (A-D) Dorsal (A, B) or lateral (C, D) views of the trunk of an E9.5 control (A, C) or *Tfap2a*/*Tfap2b* DCM (B, D) mouse embryo processed for β-gal staining (as in Fig 4A–4D). (E, F)Transverse immunofluorescent cryosections through the trunk of an E9.5 control (E) or *Tfap2a/Tfap2b* DCM (F) embryo immunostained with an anti:β-gal antibody (green), revealing migrating neural crest cells (tissue counter stained with Draq5, blue). (G, H) Lateral trunk views of E9.5 control (G) and *Tfap2a/Tfap2b* DCM (H) embryos processed by *in situ* hybridization with a *Sox10* riboprobe. Abbreviations: DCM, double conditional mutant; nt, neural tube. Scale bars = 500μM.
(PDF)

S10 Fig Assessment of additional trunk neural crest derivatives in *Tfap2a/Tfap2b* DCM embryos. (A-H) Transverse (A-D) or ventral (E-H) trunk views of E12.5 control (A, E), *Tfap2a* SCM (B, F), *Tfap2b* SCM (C, G), or *Tfap2a/Tfap2b* DCM (D, H) mouse embryos processed for β-galactosidase (β-gal) staining, labeling neural crest cells and their derivatives (as in Fig 4A–4D). (A-D) highlights the dorsal root ganglia while (E-H) highlights a portion of the enteric nervous system (ENS) populating the gastrointestinal (GI) tract. (I, J) Transverse cryosections through the GI-tract of an E12.5 control (I) or *Tfap2a/Tfap2b* DCM (J) embryo in which a fluorescent Tomato-reporter (*rTMr*) has been incorporated, labeling neural crest cells contributing to the ENS. (K, L) Late embryonic stage (E17.5) control (K) or *Tfap2a/Tfap2b* DCM (L) intestines processed for β-gal staining, revealing neural crest-derived ENS components (note, the ENS includes ‘striated’ surface staining in (K) whereas internal β-gal staining in (L) is background). Abbreviations: DCM, double conditional mutant; drg, dorsal root ganglia; SCM, single conditional mutant (A = *Tfap2a*, B = *Tfap2b*). Scale bars = 500μM.
(PDF)

S1 Table Microarray data comparing gene expression in wildtype versus *tfap2a*^*-/-*^ mutants, filtered for transcripts with unique Ensembl IDs. (XLSX)

S2 Table Microarray expression values for 20 genes annotated at ZFIN as “melanoblast”, “melanocyte”, or “pigment cell”. (XLSX)

S3 Table Genes with significant expression changes in mouse melan-a cells treated with siRNA against *Tfap2a* (si5 or siA) versus control siRNA, fold change threshold ±1.4 fold. (XLSX)

S4 Table Coordinates of TFAP2A peaks from human primary melanocytes that intersect with TFAP2A peaks from mouse melan-a cells lifted over to hg19. (XLSX)

S5 Table Coordinates of TFAP2A peaks from mouse melan-a cells that intersect with TFAP2A peaks from human primary melanocytes lifted over to mm9. (XLSX)

S6 Table Concordance of TFAP2A ChIP-seq peaks in human primary melanocytes and mouse melan-a cells. (XLSX)

S7 Table Coordinates of active peaks for TFAP2A in human and mouse, human MITF, and human TFAP2A/MITF overlap, along with the list of associated genes. (XLSX)

S8 Table List of significantly affected genes from mouse melan-a microarrays and associated mouse TFAP2A peaks. (XLSX)

S9 Table Panther GO term analysis of genes associated with active peaks of both TFAP2A and MITF. (XLSX)

S10 Table Coordinates of super-enhancers identified in human primary melanocytes and associated genes. (XLSX)

S11 Table Primer sequences for qRT-PCR and ChIP-qPCR experiments. (XLSX)
