# Supplementary figures and images for: TFAP2 paralogs regulate melanocyte differentiation in parallel with MITF
Source: PLoS Genet. 2017 Mar 1;13(3):e1006636. doi: 10.1371/journal.pgen.1006636 (PMC5352137; doi:10.1371/journal.pgen.1006636)

*tfap2a*<sup>+/-</sup>

*tfap2a*<sup>-/-</sup>

**A**

28 hpf

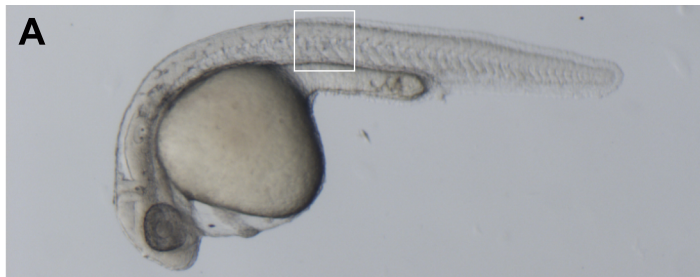

**G**

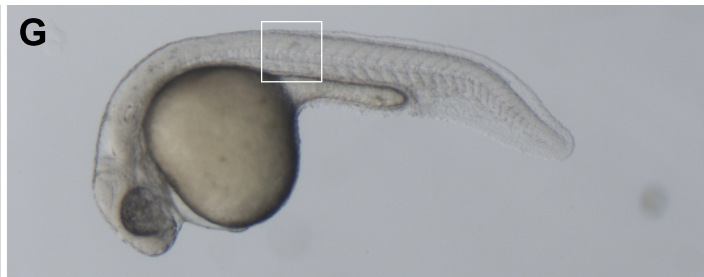

**B**

30 hpf

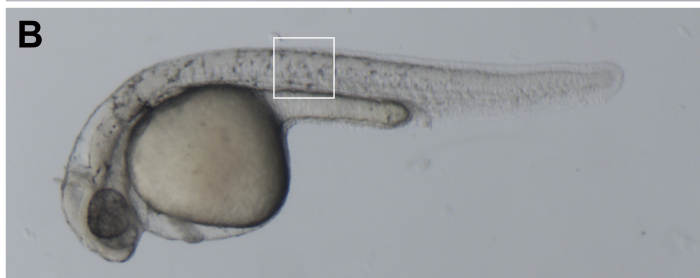

**H**

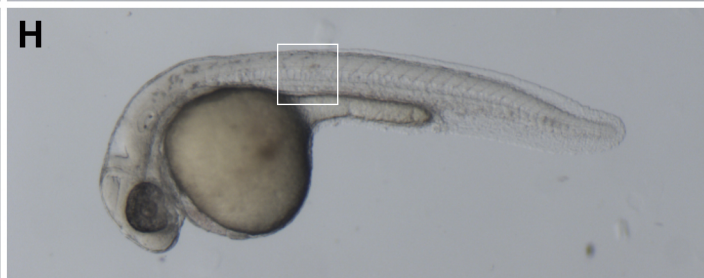

**C**

32 hpf

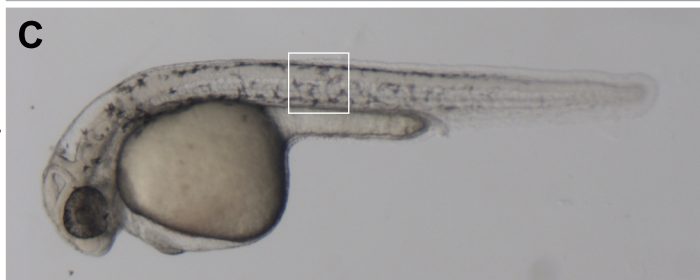

**I**

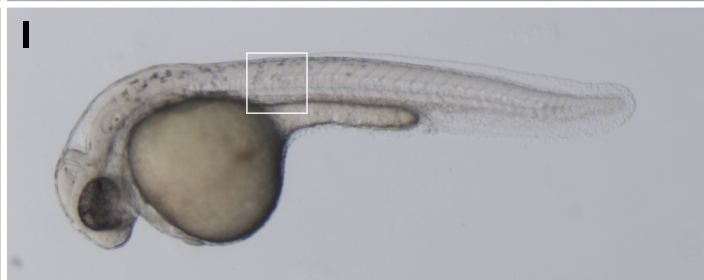

**D**

34 hpf

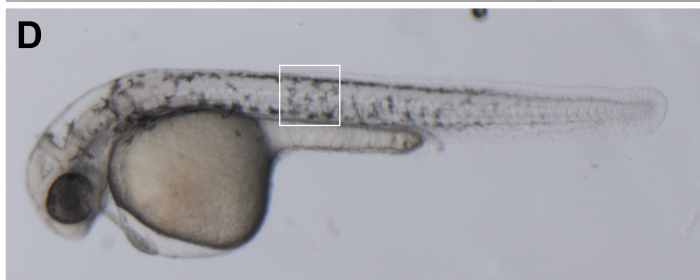

**J**

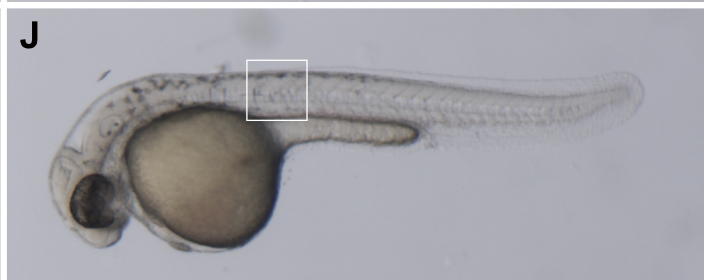

**E**

36 hpf

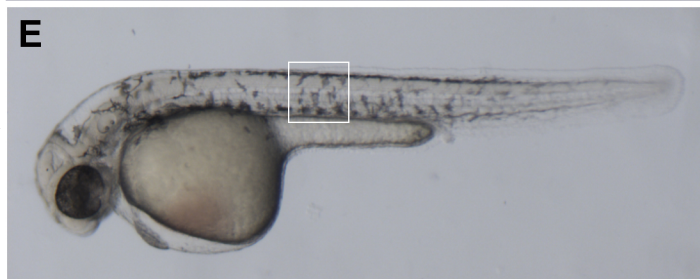

**K**

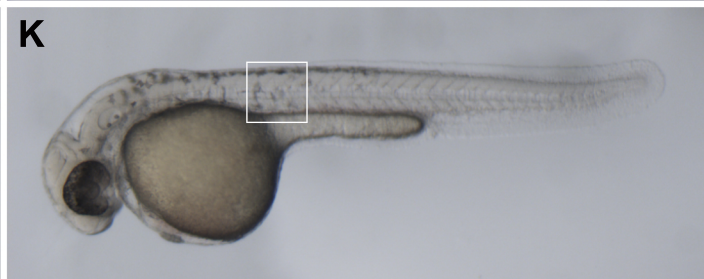

**F**

38 hpf

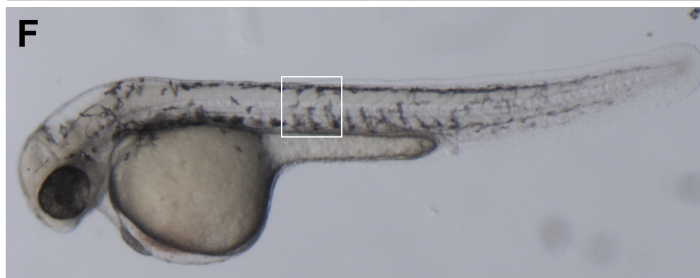

**L**

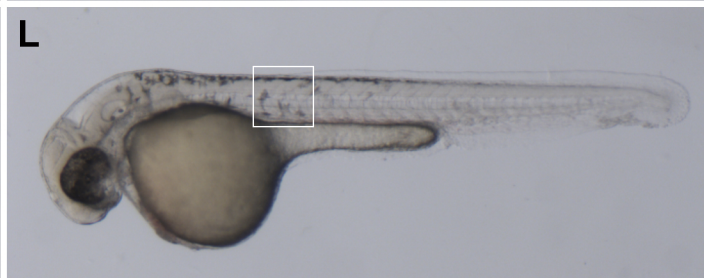

Supplement: S1 Fig — (A-L) Lateral views of live tfap2a+/- (left column) and tfap2a-/- (right column) zebrafish embryos at the indicated age from 28-38 hpf. (C, I) Melanocytes in a tfap2a+/- zebrafish (C) appear darkly pigmented by 32 hpf, whereas melanocytes at the same location in a tfap2a-/- mutant (I) at this stage remain pale and punctate. (F, L) At 38 hpf, there is still a detectable difference in the level of pigmentation in tfap2a+/- (F) and tfap2a-/- (L) mutant animals. (PDF) [file pgen.1006636.s001.pdf]

# TFAP2A Immunostaining

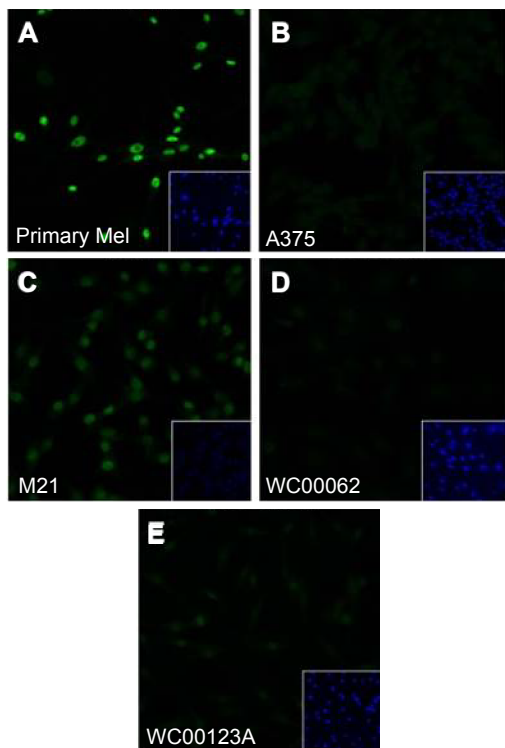

Supplement: S2 Fig — (A-E) Nuclear anti-TFAP2A immunoreactivity (green fluorescence) in human primary melanocytes (A) and four human melanoma cell lines (B-E). Inset shows DAPI counter-stain for nuclei. TFAP2A expression is low but detectable in M21s (C). (PDF) [file pgen.1006636.s002.pdf]

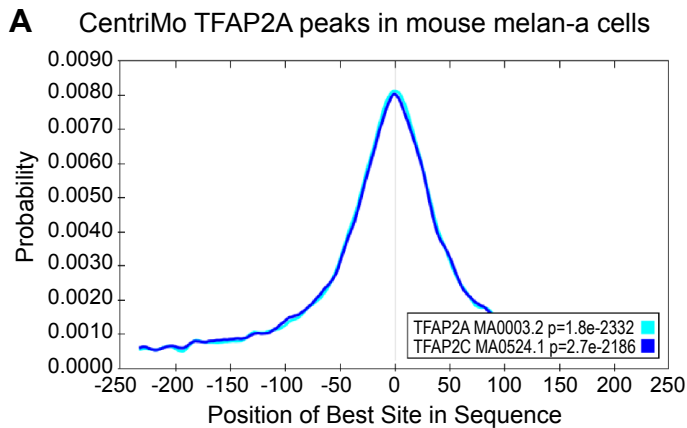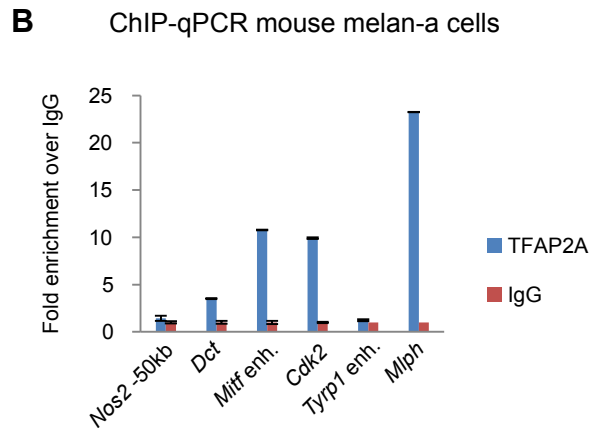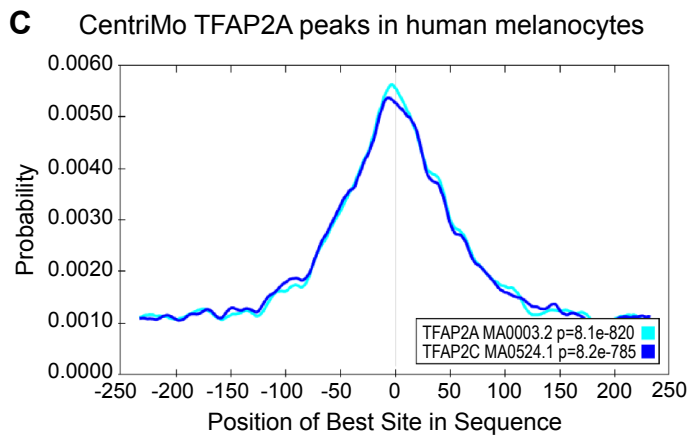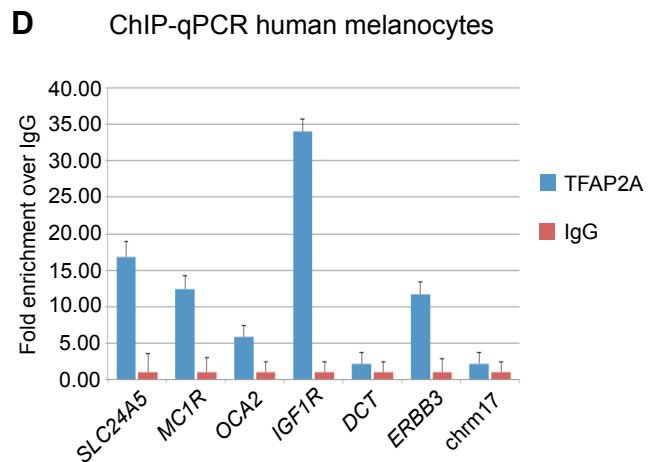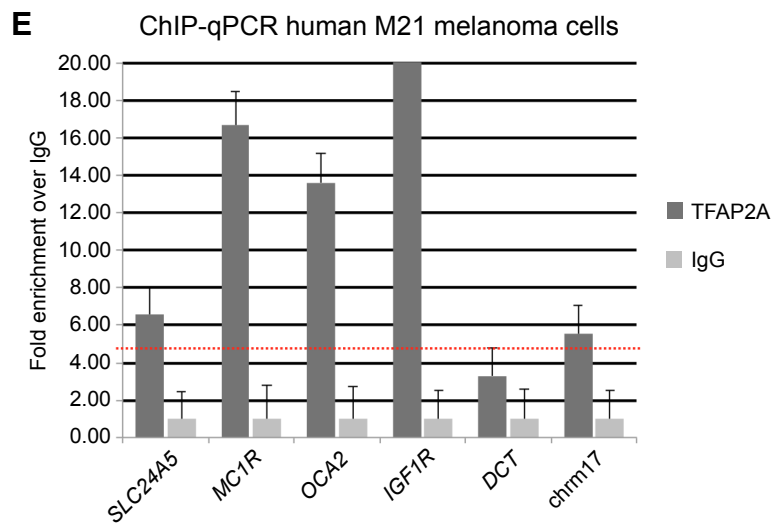

Supplement: S3 Fig — (A, B) Validation of TFAP2A ChIP-seq in mouse melan-a cells. (A) CentriMo analysis shows that mouse TFAP2A ChIP-seq peaks are centered on the TFAP2A/TFAP2C binding motif (TFAP2A p = 1.8e-2332, TFAP2C p = 2.7e-2186) (B) Four of five sites tested by ChIP-qPCR confirmed enrichment of TFAP2A binding over the IgG control, whereas an off-target site (Nos2 -50kb) showed no enrichment. (C, D) Validation of TFAP2A ChIP-seq in human primary melanocytes. (C) CentriMo analysis shows that human TFAP2A ChIP-seq peaks are centered on the TFAP2A/TFAP2C binding motif (TFAP2A p = 8.1e-820, TFAP2C p = 8.2e-785). (D) Five of six sites tested by ChIP-qPCR confirmed enrichment of TFAP2A binding over the IgG control, whereas an off-target site (chrm17) showed no enrichment. (E) Validation of TFAP2A ChIP-seq in M21 melanoma cells. Four of five sites tested by ChIP-qPCR confirmed enrichment of TFAP2A binding over the IgG control, whereas an off-target site (chrm17) showed no enrichment. Notably, DCT showed enrichment for TFAP2A binding in the mouse melan-a cells, but not in human primary melanocytes or M21s, which is consistent with the ChIP-seq results for mouse and human, respectively. (PDF) [file pgen.1006636.s003.pdf]

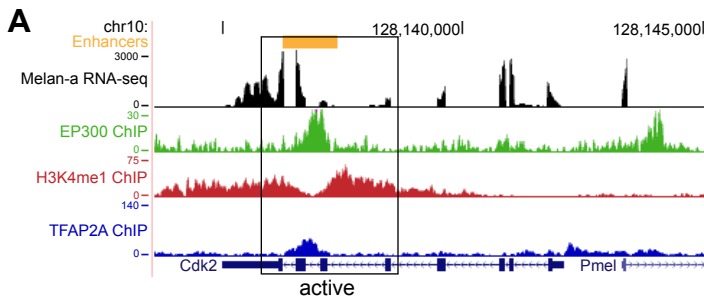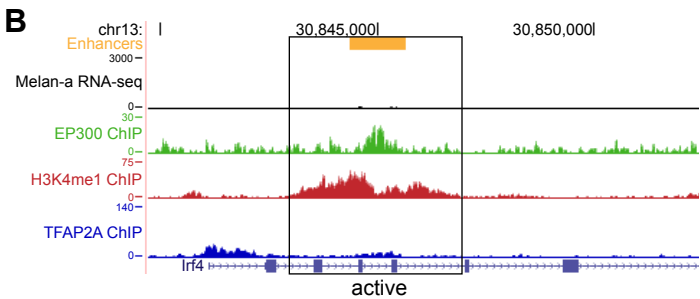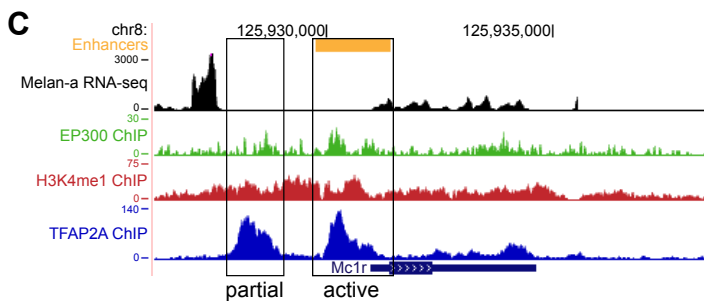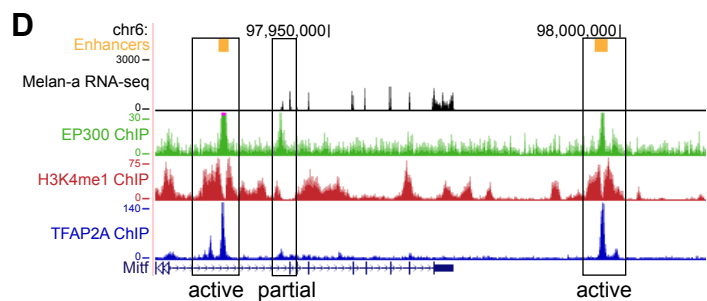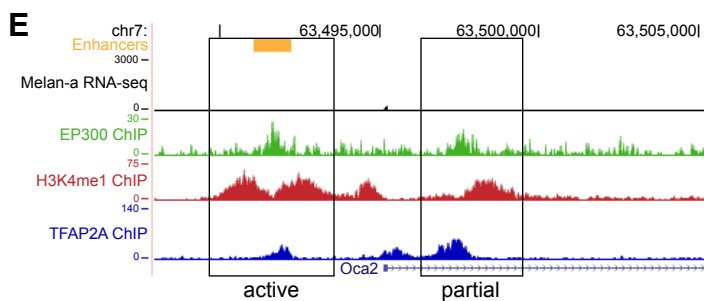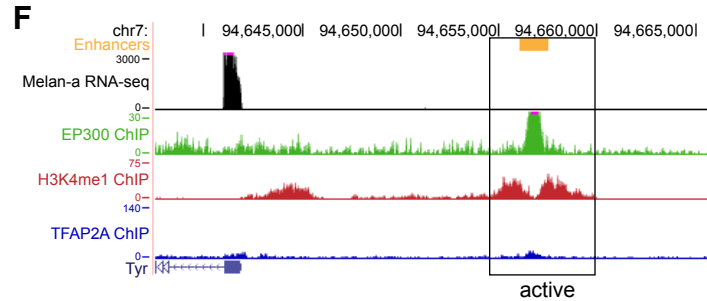

Supplement: S5 Fig — (A-F) UCSC genome browser tracks showing mouse TFAP2A ChIP-seq peaks that overlap an active enhancer signature (p300 flanked by H3K4me1) or a partial enhancer signature near the promoters of (A) Pmel, (B) Irf4, (C) Mc1r, (D) Mitf, (E) Oca2, and (F) Tyr. (PDF) [file pgen.1006636.s005.pdf]

**A**

**Human Gene Ontology Terms 13,690 TFAP2A peaks**

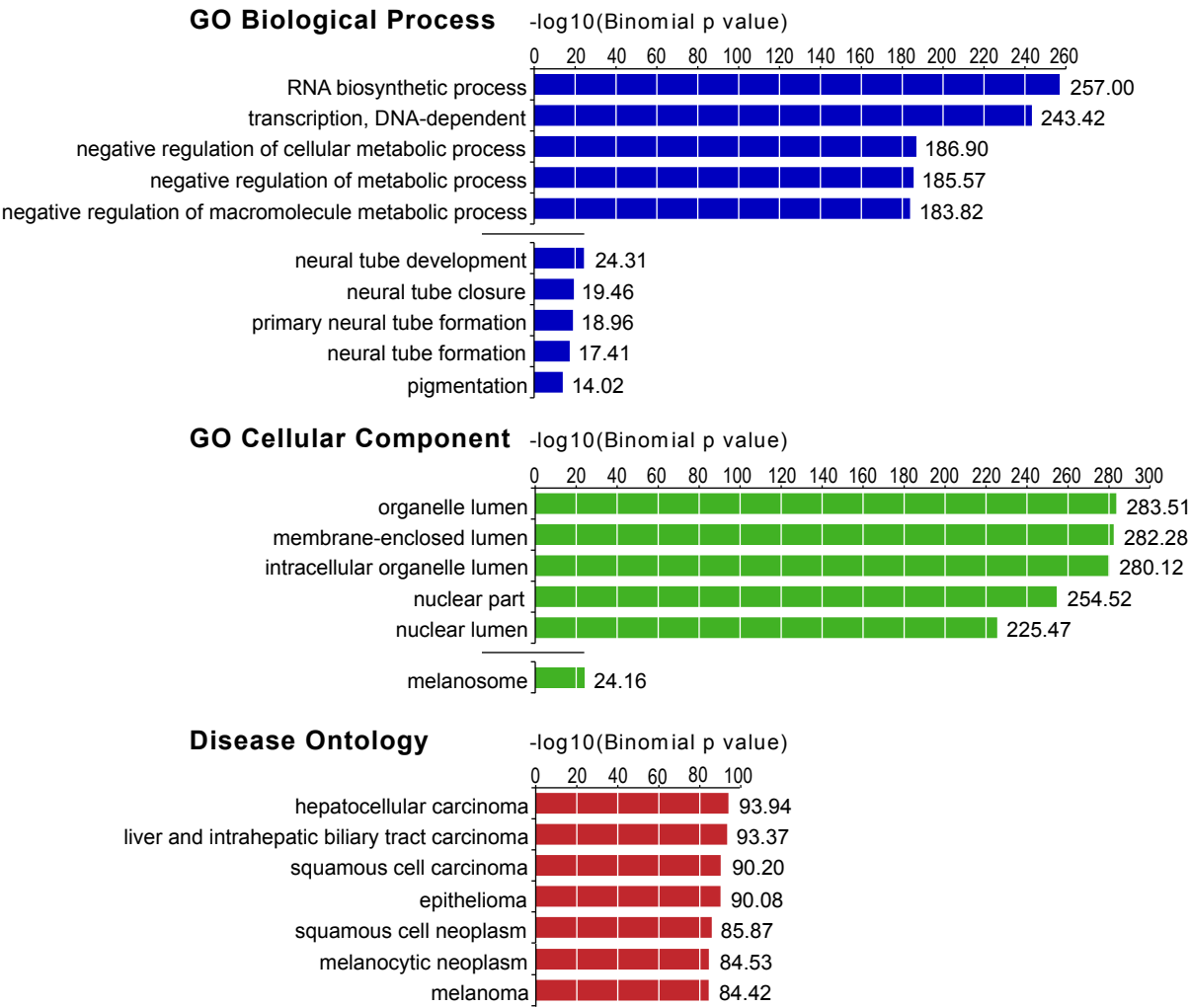

## B Mouse Gene Ontology Terms 16,305 TFAP2A peaks

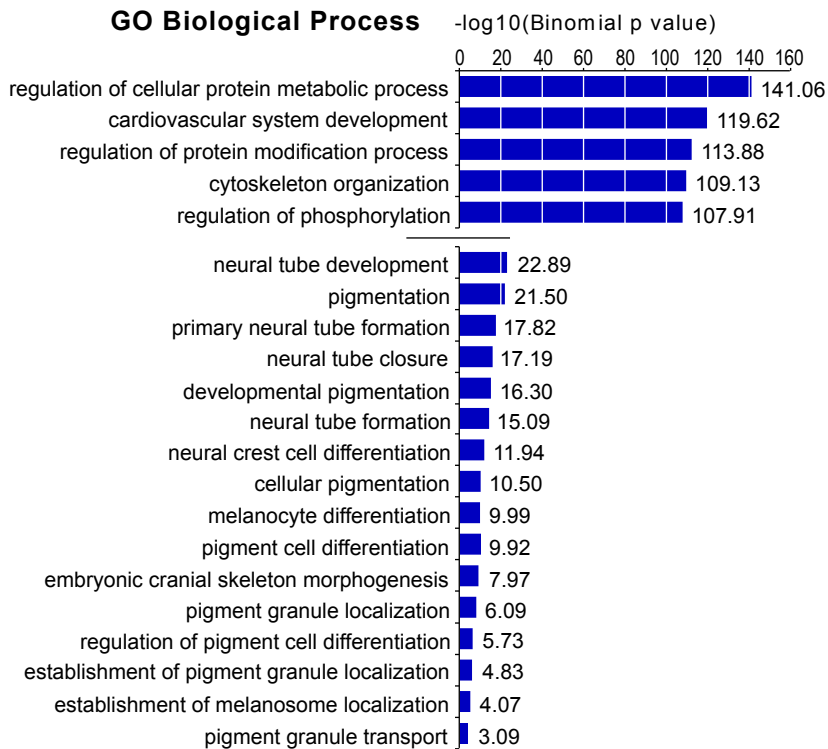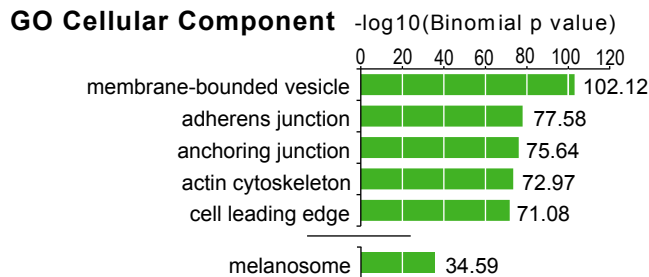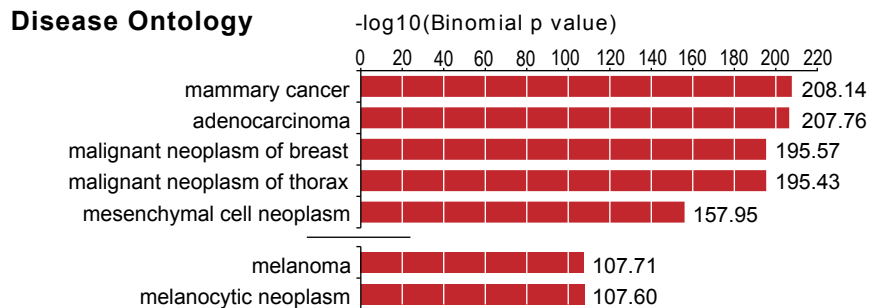

**C**

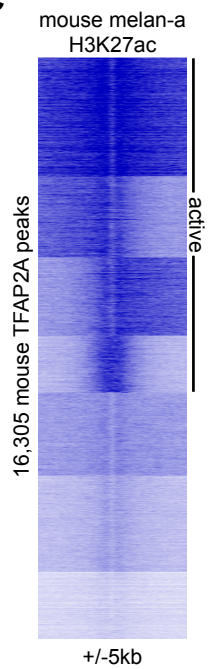

Supplement: S6 Fig — (A, B) GO term enrichment analysis of genes associated with (A) 13,690 TFAP2A peaks in human melanocytes and (B) 16,305 TFAP2A peaks in mouse melanocytes. Gene association was identified using the GREAT algorithm with an assignment rule of basal plus extension, proximal TSS -5/+1kb, distal up to 100kb. For each category, the top five most significant terms are listed, followed by select terms of interest. Significance is charted as -log10(binomial p-value). (C) Density-based clustering of H3K27ac signal at TFAP2A peaks in mouse melanocytes (H3K27ac data from [14]). (PDF) [file pgen.1006636.s006.pdf]

# human melanocyte super-enhancer

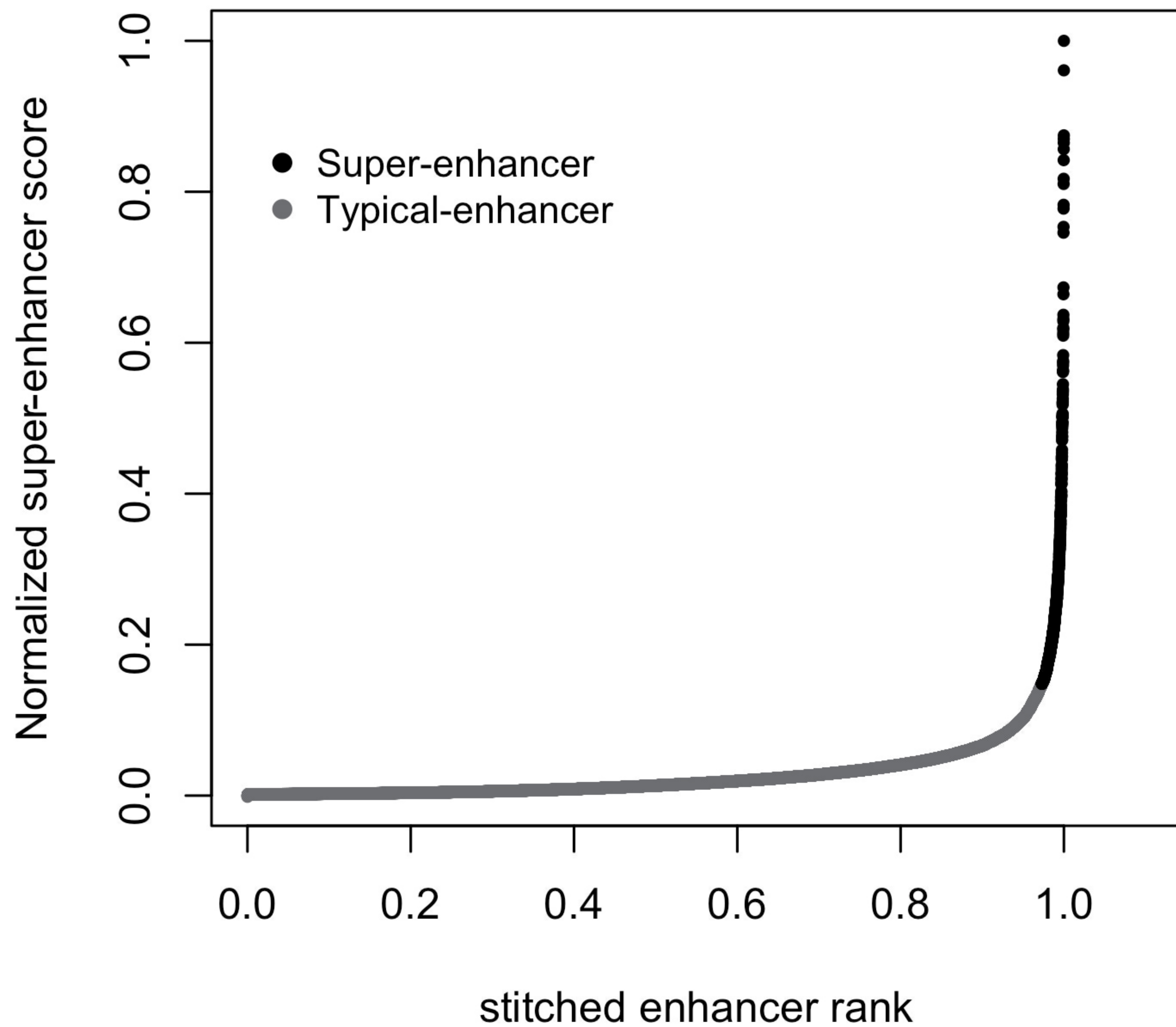

Supplement: S7 Fig — Chart depicting typical enhancers (gray) and super-enhancers (black) in human melanocytes. (PDF) [file pgen.1006636.s007.pdf]

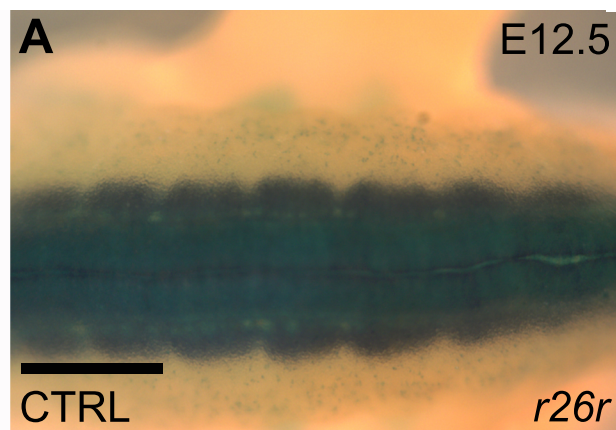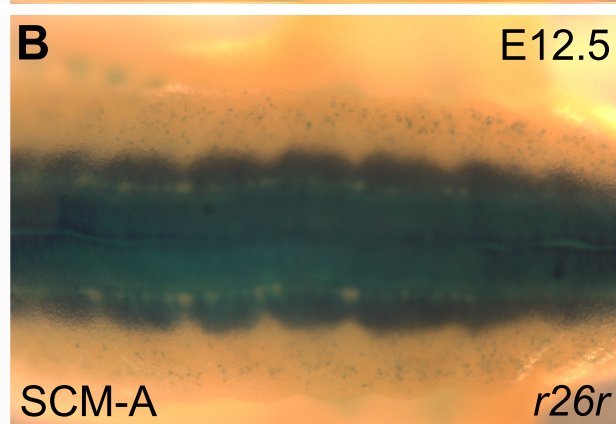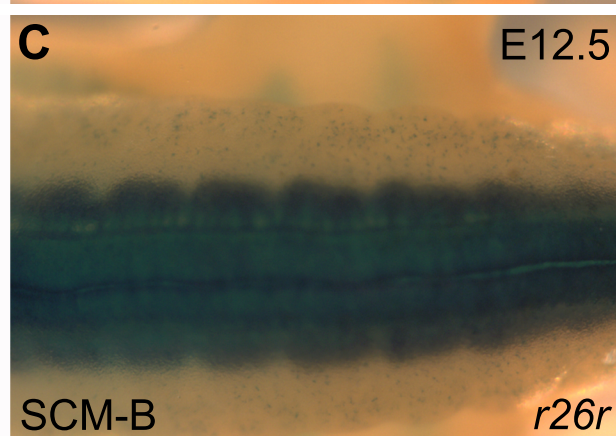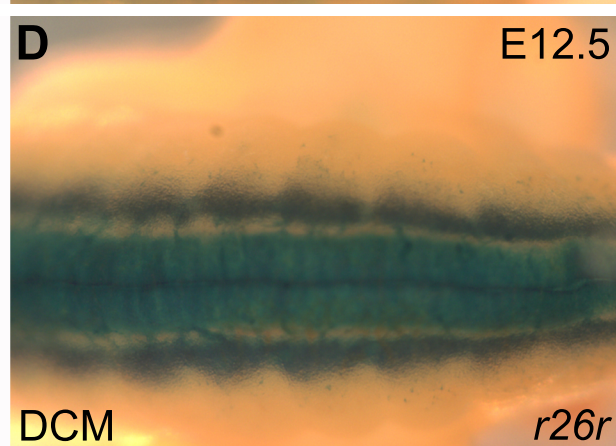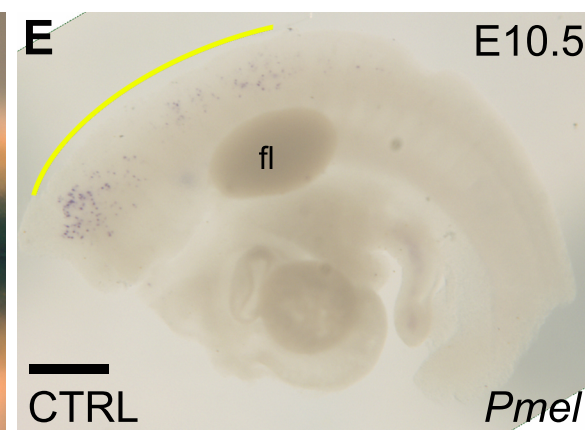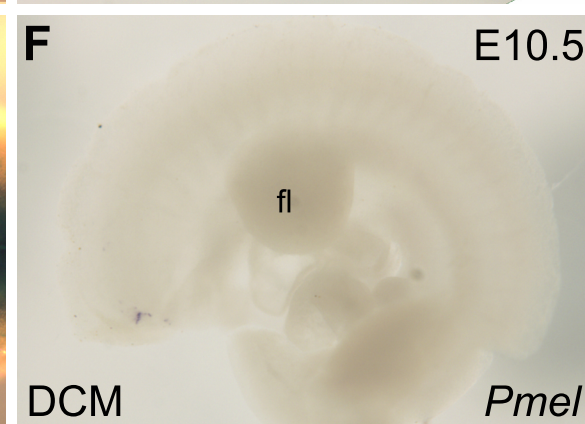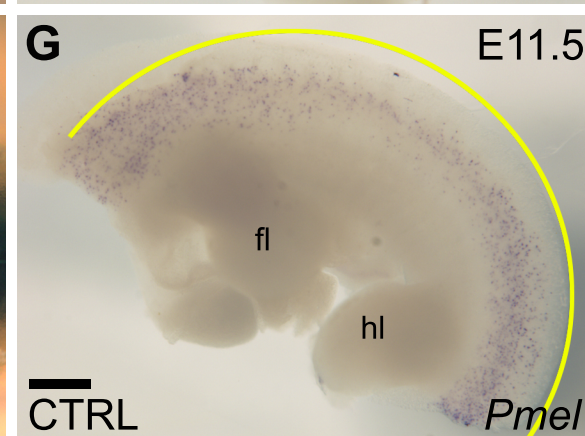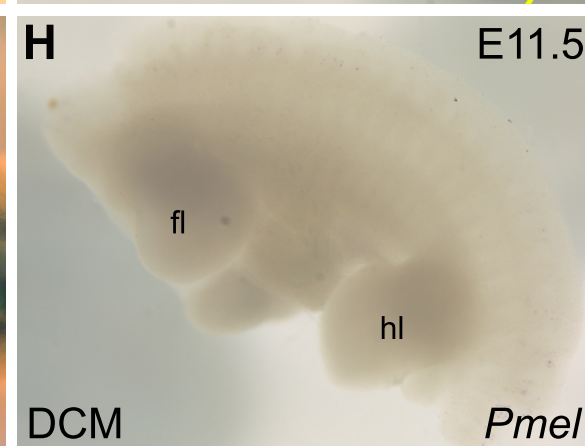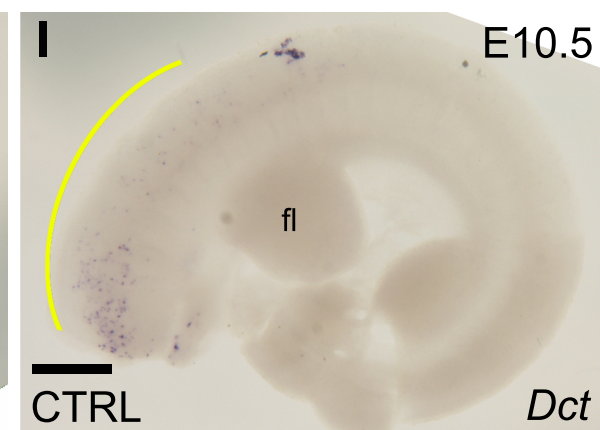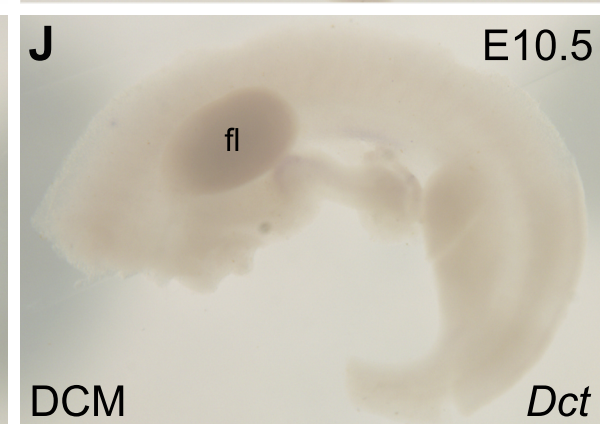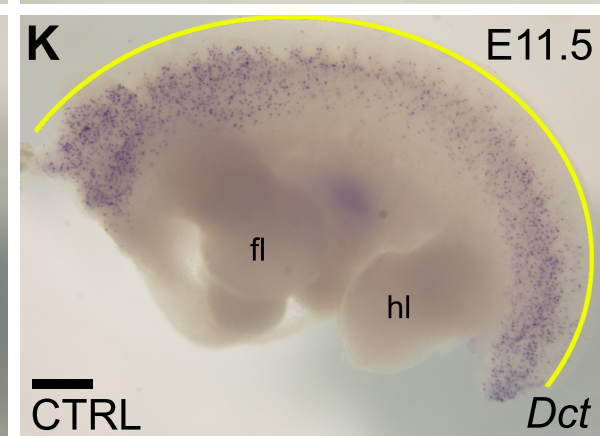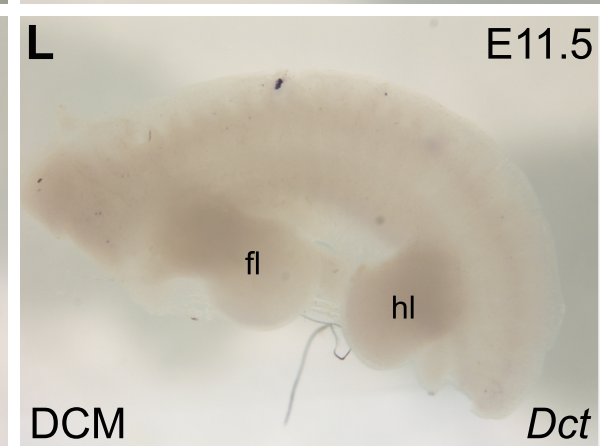

Supplement: S8 Fig — (A-D) Dorsal views of E12.5 control (A), Tfap2a SCM (B), Tfap2b SCM (C), or Tfap2a/Tfap2b DCM (D) mouse embryos processed for β-galactosidase (β-gal) staining, labeling neural crest cells and their derivatives, including melanocytes migrating on the ventrolateral pathway (as in Fig 4A–4D). (E-L) Lateral views of E10.5 control (E, I) or Tfap2a/Tfap2b DCM (F, J) and E11.5 control (G, K) or Tfap2a/Tfap2b DCM (H, L) mouse embryos processed for Pmel (E-H) or Dct (I-L) expression by in situ hybridization. Yellow lines (in E, I, G, K) indicate extent of rostral-caudal regions of in situ signal, rostral left. Abbreviations: DCM, double conditional mutant; fl, forelimb; hl, hindlimb; SCM, single conditional mutant (A = Tfap2a or B = Tfap2b). Scale bars = 500μM. (PDF) [file pgen.1006636.s008.pdf]

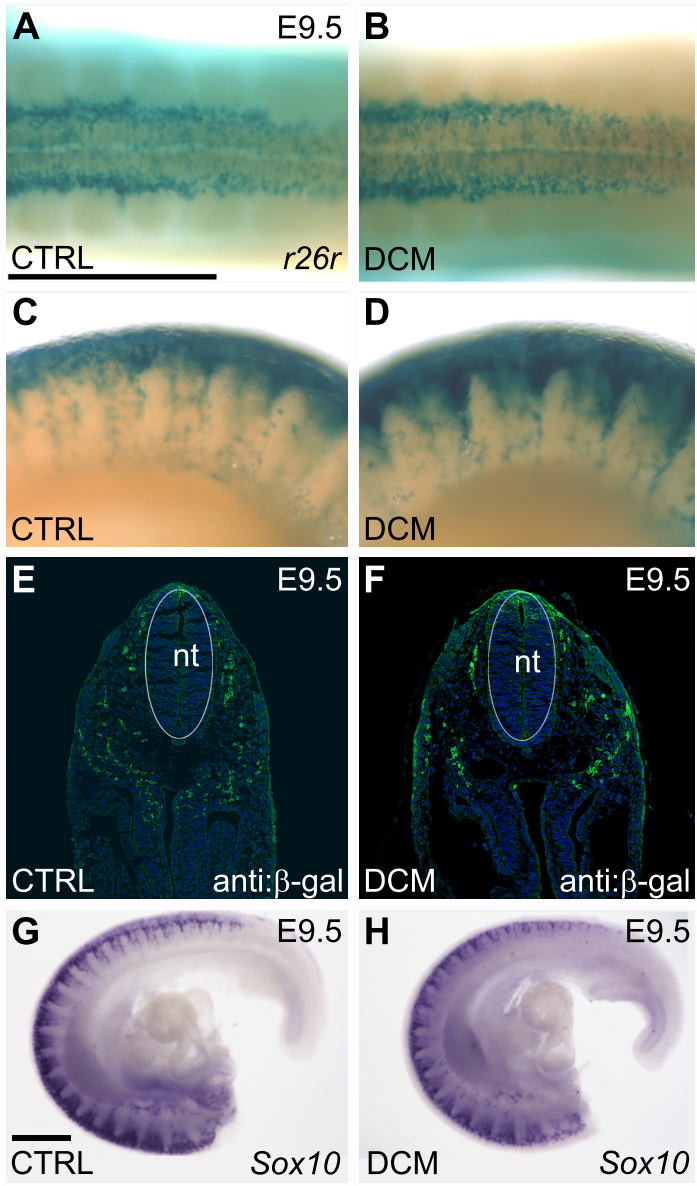

Supplement: S9 Fig — (A-D) Dorsal (A, B) or lateral (C, D) views of the trunk of an E9.5 control (A, C) or Tfap2a/Tfap2b DCM (B, D) mouse embryo processed for β-gal staining (as in Fig 4A–4D). (E, F)Transverse immunofluorescent cryosections through the trunk of an E9.5 control (E) or Tfap2a/Tfap2b DCM (F) embryo immunostained with an anti:β-gal antibody (green), revealing migrating neural crest cells (tissue counter stained with Draq5, blue). (G, H) Lateral trunk views of E9.5 control (G) and Tfap2a/Tfap2b DCM (H) embryos processed by in situ hybridization with a Sox10 riboprobe. Abbreviations: DCM, double conditional mutant; nt, neural tube. Scale bars = 500μM. (PDF) [file pgen.1006636.s009.pdf]

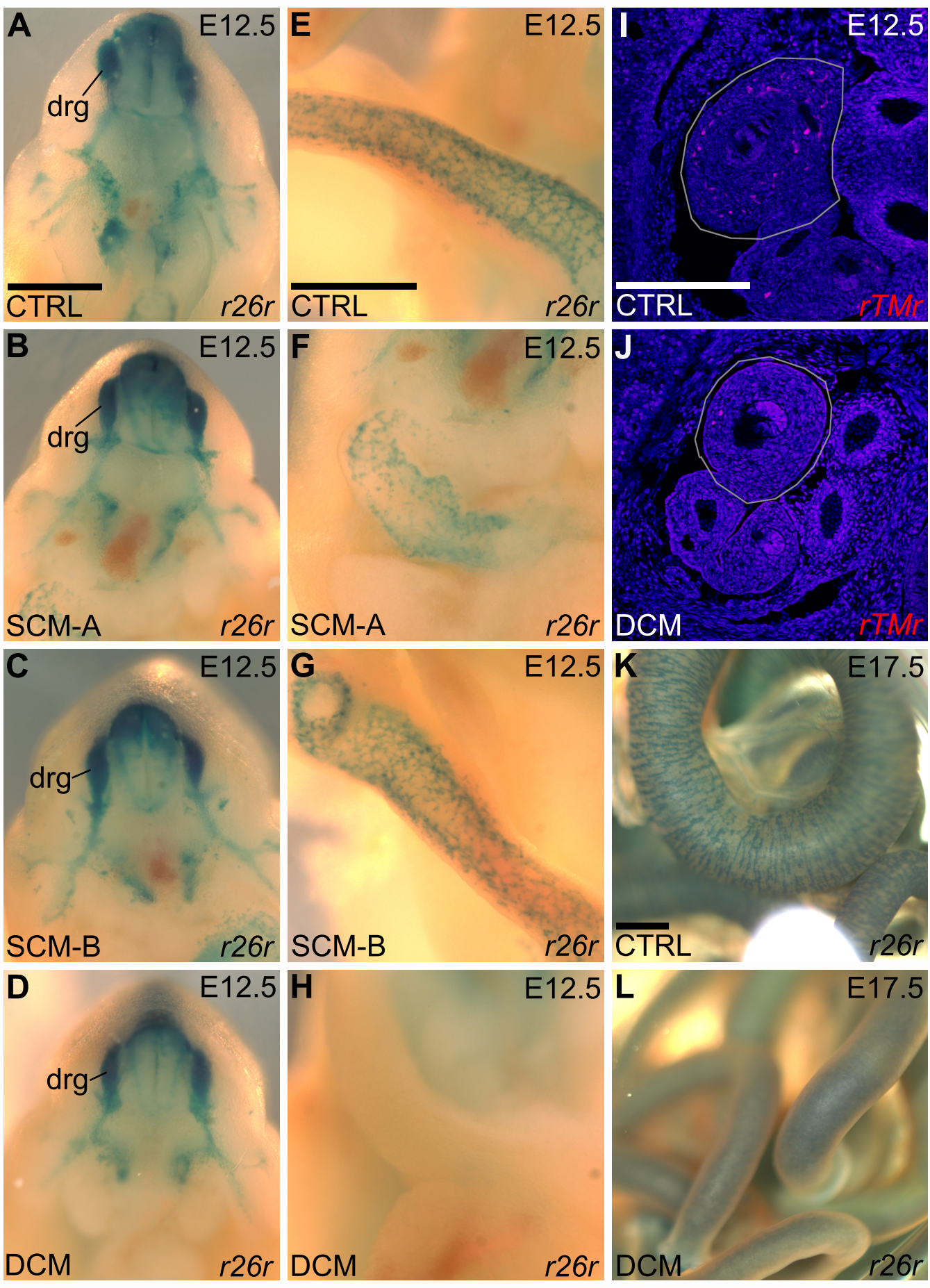

Supplement: S10 Fig — (A-H) Transverse (A-D) or ventral (E-H) trunk views of E12.5 control (A, E), Tfap2a SCM (B, F), Tfap2b SCM (C, G), or Tfap2a/Tfap2b DCM (D, H) mouse embryos processed for β-galactosidase (β-gal) staining, labeling neural crest cells and their derivatives (as in Fig 4A–4D). (A-D) highlights the dorsal root ganglia while (E-H) highlights a portion of the enteric nervous system (ENS) populating the gastrointestinal (GI) tract. (I, J) Transverse cryosections through the GI-tract of an E12.5 control (I) or Tfap2a/Tfap2b DCM (J) embryo in which a fluorescent Tomato-reporter (rTMr) has been incorporated, labeling neural crest cells contributing to the ENS. (K, L) Late embryonic stage (E17.5) control (K) or Tfap2a/Tfap2b DCM (L) intestines processed for β-gal staining, revealing neural crest-derived ENS components (note, the ENS includes ‘striated’ surface staining in (K) whereas internal β-gal staining in (L) is background). Abbreviations: DCM, double conditional mutant; drg, dorsal root ganglia; SCM, single conditional mutant (A = Tfap2a, B = Tfap2b). Scale bars = 500μM. (PDF) [file pgen.1006636.s010.pdf]
