# Supplementary material for: TFAP2 paralogs regulate melanocyte differentiation in parallel with MITF
Source: PLoS Genet. 2017 Mar 1;13(3):e1006636. doi: 10.1371/journal.pgen.1006636 (PMC5352137; doi:10.1371/journal.pgen.1006636)
Supplement: S4 Fig — (A) Pie chart showing distribution of human TFAP2A peaks with respect to genomic features. TSS, transcription start site; TTS, transcription termination site. (B) Distance from TSS to the nearest TFAP2A peak for genes in three expression categories: highest 1000, median 1000, or lowest 1000. Highly expressed genes are more likely to have promoter-proximal TFAP2A peaks. Human expression data from the Roadmap Epigenomics Project GSM958174 [64]. (PDF) [file pgen.1006636.s004.pdf]

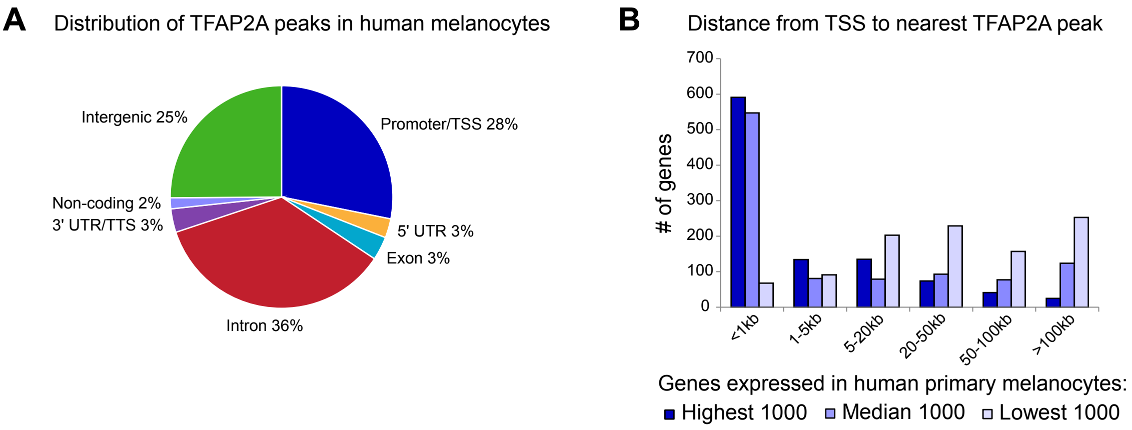
A
Distribution of TFAP2A peaks in human melanocytes
Intergenic 25%
Promoter/TSS 28%
Non-coding 2%
3' UTR/TTS 3%
5' UTR 3%
Exon 3%
Intron 36%
B
Distance from TSS to nearest TFAP2A peak
# of genes
700
600
500
400
300
200
100
0
<1kb
1-5kb
5-20kb
20-50kb
50-100kb
>100kb
Genes expressed in human primary melanocytes:
Highest 1000
Median 1000
Lowest 1000
